# PPARγ phase separates with RXRα at PPREs to regulate target gene expression

**DOI:** 10.1038/s41421-022-00388-0

**Published:** 2022-04-26

**Authors:** Zhean Li, Lingling Luo, Wenxia Yu, Ping Li, Danfeng Ou, Jia Liu, Hanhui Ma, Qinhu Sun, Aibin Liang, Cheng Huang, Tian Chi, Xingxu Huang, Yu Zhang

**Affiliations:** 1 School of Life Science and Technology, ShanghaiTech University, Shanghai, China; 2 Shanghai Institute for Advanced Immunochemical Studies, ShanghaiTech University, Shanghai, China; 3 School of Pharmacy, Shanghai University of Traditional Chinese Medicine, Shanghai, China; 4 Department of Hematology, Tongji Hospital of Tongji University, Shanghai, China; 5 International Academy of Optoelectronics at Zhaoqing, South China Normal University, Zhaoqing, Guangdong, China; 6 Shanghai R&D Center for Standardization of Chinese Medicines, Shanghai, China; 7 Zhejiang Provincial Key Laboratory of Pancreatic Disease, The First Affiliated Hospital, and Institute of Translational Medicine, Zhejiang University School of Medicine, Hangzhou, Zhejiang, China

**Keywords:** Transcriptional regulatory elements, Protein aggregation

## Abstract

Peroxisome proliferator-activated receptor (PPAR)-γ is a key transcription activator controlling adipogenesis and lipid metabolism. PPARγ binds PPAR response elements (PPREs) as the obligate heterodimer with retinoid X receptor (RXR) α, but exactly how PPARγ orchestrates the transcriptional response is unknown. This study demonstrates that PPARγ forms phase-separated droplets in vitro and solid-like nuclear condensates in cell, which is intriguingly mediated by its DNA binding domain characterized by the zinc finger motif. Furthermore, PPARγ forms nuclear condensates at PPREs sites through phase separation to compartmentalize its heterodimer partner RXRα to initiate PPARγ-specific transcriptional activation. Finally, using an optogenetic approach, the enforced formation of PPARγ/RXRα condensates leads to preferential enrichment at PPREs sites and significantly promotes the expression of PPARγ target genes. These results define a novel mechanism by which PPARγ engages the phase separation principles for efficient and specific transcriptional activation.

## Introduction

Peroxisome proliferator-activated receptors (PPARs) are a group of nuclear receptor superfamily proteins that function as ligand-activated transcription factors involved in diverse roles in cellular differentiation, development, and metabolism. PPARs heterodimerize with retinoid X receptor (RXR) to regulate the expression of a cluster of genes by binding to PPAR response elements (PPREs), which have been identified in the promoters of genes involved in adipogenesis, lipid and glucose metabolism, and homeostasis^1^. The PPAR family consists of three isoforms: PPARα (NR1C1), PPARβ/δ (NR1C2), and PPARγ (NR1C3). PPAR isoforms are structurally homologous, comprising an N-terminal transactivation domain (AF1), a DNA binding domain (DBD) and a C-terminal ligand-binding domain (LBD, containing a ligand-dependent transactivation function (AF2))^2^. PPARα mainly mediates energy homeostasis, PPARβ/δ activation promotes fatty acid metabolism, and PPARγ is a dominant regulator of obesity and insulin resistance. They have been well identified as drug targets for the treatment of metabolic syndrome^2–6^.

PPAR isoforms are encoded separately and have distinct functions but often act as a functional group to coordinate cellular processes^7^. PPARγ, the best-studied member of this family, serves as a master regulator of adipocyte differentiation, insulin resistance, and inflammation through transcriptional activation. Synthetic ligands, such as thiazolidinediones (TZDs), are potent insulin sensitizers that induce PPARγ activation. However, the TZD class of drugs has been contraindicated in patients due to the significant adverse effects with weight gain and fluid retention induced by chronic PPARγ activation^1,6,8^. Accordingly, further understanding of how TZDs trigger robust PPARγ activation, as well as alternative approaches for regulating PPARγ signaling, will potentially provide improved therapies for insulin resistance.

Genomic studies of PPARγ have demonstrated that comprehensive binding sites are mainly distributed in introns (45%) and intergenic enhancers (48%). Intriguingly, although PPARγ binding sites are rare in the promoters of genes (accounting for only 3% of all binding sites), PPRE-mediated transcription is proposed as a primary mechanism for PPARγ regulatory function^6,9–11^. Little is known regarding the precise mechanism by which PPRE mediates the function of PPARγ. Recent studies suggest that assembly of the transcription machinery at specific genomic sites occurs through the formation of liquid-like or solid-like transcriptional condensates. In particular, the condensates compartmentalize and concentrate the transcription regulators to achieve transcription initiation and control^12–19^. Given the above-mentioned studies of the PPRE-mediated transcription regulation by PPARγ, we investigated whether PPARγ prefers to form phase-separated condensates at PPREs to regulate the expression of its target genes.

Here, we report that the transcription factor PPARγ phase separates with its heterodimer partner RXRα to form nuclear condensates, and this process is mediated by its DBD instead of an intrinsically disordered region (IDR), a type of region that is known to be a driver of phase separation^20–22^. Furthermore, PPARγ prefers to form nuclear condensates at the PPRE sites to compartmentalize RXRα to initiate the transcription of PPARγ-targeted genes involved in adipogenesis. Finally, enforced formation of PPARγ/RXRα condensates at PPRE sites significantly promoted target gene expression using optogenetic experiments. These results define a novel framework to account for PPRE-mediated transcriptional activation by PPARγ and provide an alternative approach for efficient and specific transcriptional activation through phase separation.

## Results

### PPARγ compartmentalizes RXRα to form phase-separated condensates in vitro and in the nucleus

**Fig. 1:**
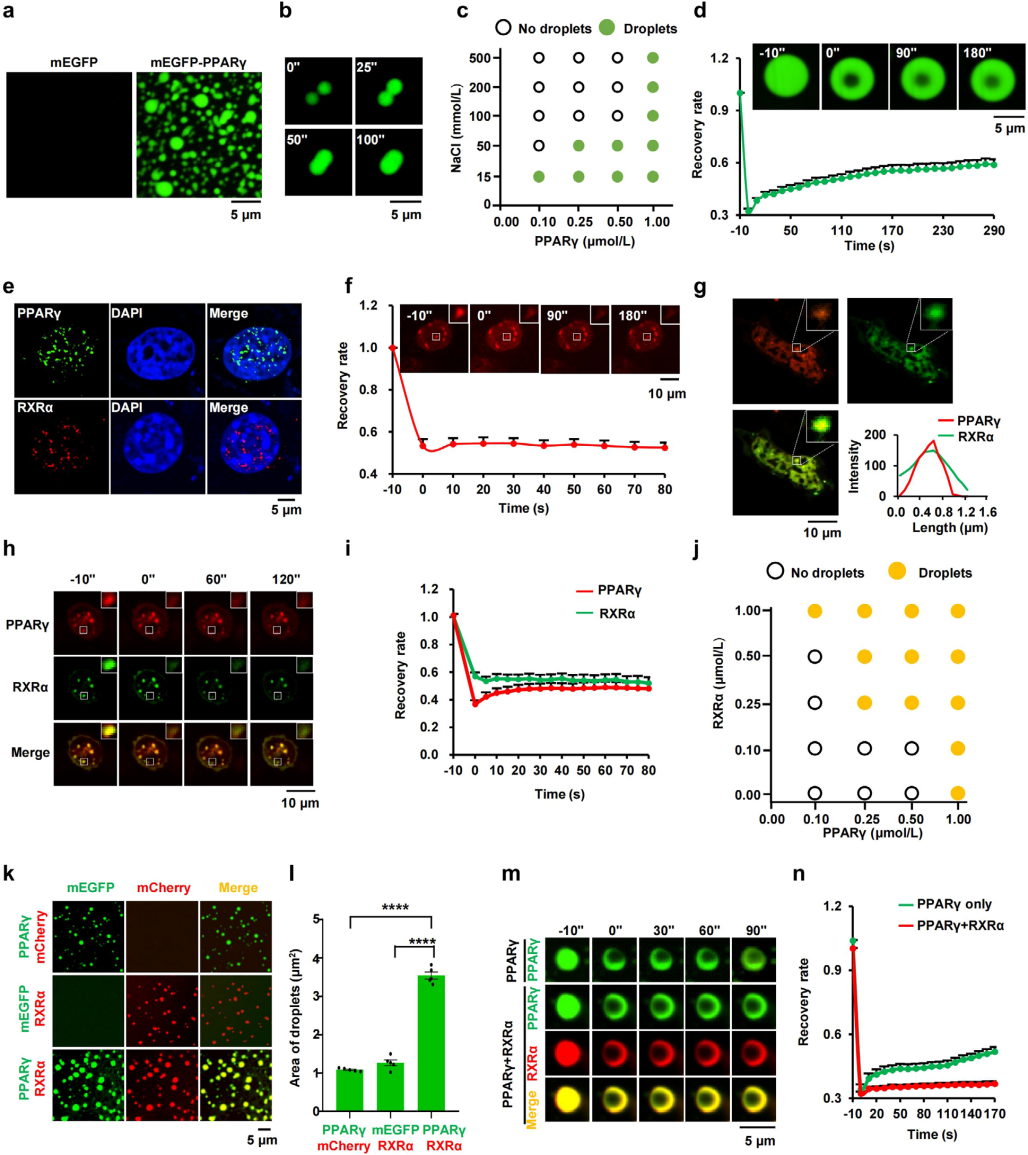
PPARγ phase separated with RXRα in vitro and in cells. **a** Representative images of droplet formation as indicated (5 μmol/L protein, 150 μmol/L NaCl, 10% PEG-8000). **b** Time-lapse micrographs of merging droplets (5 μmol/L protein, 150 μmol/L NaCl, 10% PEG-8000). **c** Phase diagram of PPARγ in the presence of different concentrations of NaCl, displaying phase separation potential of the protein is dependent on salt concentration (10% PEG-8000). **d** Quantifications of changes in the fluorescence measurement of mEGFP-PPARγ droplets after photobleaching were plotted over time (10 μmol/L protein, 150 μmol/L NaCl). The background was subtracted from the fluorescence measurement. Values represent means ± SEM (*n* = 15). Scale bar, 5 μm. **e** Immunofluorescence assay for PPARγ (green) or RXRα (red) in fixed 3T3-L1 cells. Fluorescence signal is shown alone (left) or merged with DAPI stain (right). **f** FRAP recovery images and recovery curve of nuclear mCherry-PPARγ condensates. The dotted square displays the region of photobleaching. Data are shown as means ± SEM (*n* = 15). Scale bar, 10 μm. **g** Confocal images of 3T3-L1 adipocytes transfected with mCherry-PPARγ and mEGFP-RXRα for 48 h (*n* = 20). Scale bar, 10 μm. **h** FRAP analysis of PPARγ/RXRα condensates. The dotted square displays the region of photobleaching. Scale bar, 5 μm. **i** Quantification of changes in the fluorescence measurement of PPARγ/RXRα condensates after photobleaching were plotted over time. The background was subtracted from the fluorescence measurement. Values represent means ± SEM (*n* = 15). **j** Phase diagram of PPARγ in the presence of different concentrations of RXRα (150 μmol/L NaCl, 10% PEG-8000). **k** Representative fluorescence microscopy images of a mixture of mEGFP-PPARγ/mCherry, mEGFP/mCherry-RXRα and mEGFP-PPARγ/mCherry-RXRα (2 μmol/L protein, 150 μmol/L NaCl, 10% PEG-8000), respectively. Scale bar, 5 μm. **l** Column scatter charts display average droplet area of each image related to panel **k**. Data are shown as means ± SEM (*n* = 5). **m** FRAP analysis of PPARγ alone or PPARγ/RXRα droplets (5 μmol/L protein, 150 μmol/L NaCl, 10% PEG-8000). Scale bar, 5 μm. **n** Quantification of changes in the fluorescence measurement of PPARγ alone or PPARγ/RXRα droplets after photobleaching were plotted over time. The background was subtracted from the fluorescence measurement. Values represent means ± SEM (*n* = 15). One-way analysis of variance (ANOVA) for panel **l**. *P < 0.05, **P < 0.01, ***P < 0.001, ****P < 0.0001. ns, not significant.

To investigate whether phase separation could be the mechanism by which PPARγ regulates its target gene expression, we first assessed the ability of PPARγ to undergo phase separation. Recombinant mEGFP-PPARγ fusion protein was expressed in and purified from *E. coli* (Supplementary Fig. S1a). When added to a buffer containing 10% polyethylene glycol (PEG)-8000 (a molecular crowding agent), purified mEGFP-PPARγ produced opaque solution, in contrast to the mEGFP control (Supplementary Fig. S1b). The other two isoforms (PPARα and PPARβ), which share homologous DBDs and LBDs with PPARγ (Supplementary Fig. S1c), led to similar opaque solution in the presence of PEG-8000 (Supplementary Fig. S1b). Next, we detected the optical density of the PPAR isoform solution to confirm phase separation occurrence via a turbidity assay. The solutions for all three isoforms of PPAR became turbid upon PEG-8000 addition, further indicating the features of phase separation (Supplementary Fig. S1d). Fluorescence microscopy revealed that the fusion proteins existed as free-moving, micron-sized spherical droplets (Fig. 1a, b; Supplementary Fig. S1e), indicating that PPAR isoforms (PPARα, PPARβ, and PPARγ) have the ability to undergo phase separation. Phase separation of PPARγ is driven by a high protein concentration and dampened by a high sodium chloride concentration (Fig. 1c; Supplementary Fig. S1f). Furthermore, 1,6-hexanediol (1,6-HD, a molecule known to disrupt hydrophobic interaction-induced phase separation) treatment remarkably impaired the formation of PPARγ droplets (Supplementary Fig. S1g, h). These results suggest that hydrophobic and electrostatic interactions contributed to this process^23^. After photobleaching of an inner region of mEGFP-PPARγ, PPARγ showed a moderate recovery of 30% at 5 min, revealing that PPARγ droplets exhibited low inner mobility (Fig. 1d). Next, we tested whether PPARγ has the ability to form the phase-separated condensates in intact cells. Using a PPARγ-specific antibody to label endogenous PPARγ in 3T3-L1 cells, we found that the puncta became visible in the nucleus (Fig. 1e). To further determine whether such puncta appear in live cell, mCherry-labeled PPARγ was stably expressed in 3T3-L1 cells. Remarkably, live-cell fluorescence microscopy revealed that PPARγ formed discrete puncta in the nucleus, whereas the mCherry control was apparently dispersed throughout the cell (Supplementary Fig. S2a). PPARα and PPARβ showed similar features of condensates formation in cells (Supplementary Fig. S2a). Furthermore, FRAP experiments showed that PPARγ puncta were not dynamic and lacked fluid recovery upon photobleaching, suggesting that PPARγ condensates exhibit more solid-like properties, versus liquid-like properties (Fig. 1f).

RXRα, a member of the nuclear receptor superfamily, is a promiscuous partner of heterodimeric associations with PPARγ that mediates PPARγ-specific transcriptional activity. Structurally, the heterodimer interface is comprised of hydrophobic and charged residues, of which complementarity would further stabilize the heterodimer^24,25^. To assess whether RXRα partitions into PPARγ condensates, we first investigated the feature of RXRα phase separation. Similar to PPARγ, RXRα puncta were also observed in the nucleus of both fixed cell and live cell (Fig. 1e; Supplementary Fig. S2b). Moreover, PPARγ heterodimerized with RXRα to form heterotypic puncta in the nucleus, which exhibited gel or solid-like state (Fig. 1g–i). We next asked whether the heterodimerization of RXRα to PPARγ modulates phase separation of PPARγ in vitro. PPARγ failed to phase separate at the protein concentration of 0.25 μmol/L in a buffer containing 150 μmol/L NaCl and 10% PEG-8000. Co-addition of RXRα at concentration higher than 0.25 μmol/L triggered PPARγ phase separation, indicating RXRα lowered the threshold for PPARγ phase separation in a concentration-dependent manner (Fig. 1j; Supplementary Fig. S2c). Furthermore, mCherry-RXRα formed droplets similar to PPARγ in vitro and then phase separated with mEGFP-PPARγ to form heterotypic droplets that were much larger than the droplets formed by each protein alone, indicating that PPARγ/RXRα heterodimerization enhanced phase-separated condensation (Fig. 1k, l). FRAP assay showed the inner fluorescence intensity of PPARγ droplets failed to recover when PPARγ phase separated with RXRα (Fig. 1m, n). Together, these data suggest that PPARγ/RXRα heterodimers form solid-like phase-separated condensates.

### The DNA binding domain is necessary for PPARγ phase separation

**Fig. 2:**
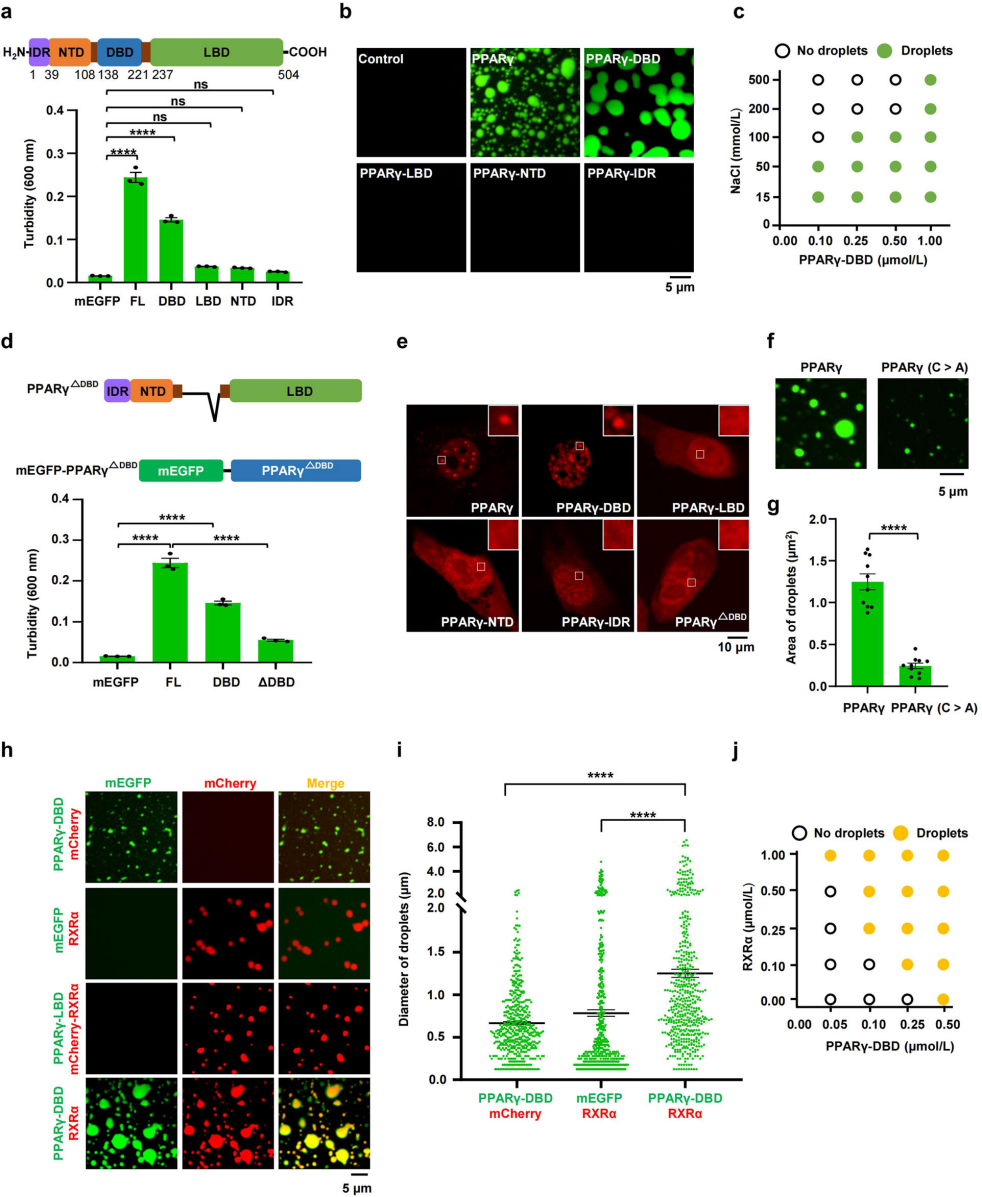
DBD is required for PPARγ phase separation. **a** Turbidity assay was used to quantify phase separation of PPARγ truncations (10 μmol/L protein, 150 μmol/L NaCl, 10% PEG-8000). OD600 was normalized to the measurement of mEGFP control (*n* = 3). Data are shown as means ± SEM. Top, a schematic representation of the PPARγ domains. **b** Representative fluorescence microscopy images of truncated forms of PPARγ-FL (10 μmol/L protein, 150 μmol/L NaCl, 10% PEG-8000). **c** Phase diagram of PPARγ-DBD in the presence of different concentrations of NaCl, displaying that phase separation potential of the protein is dependent on salt concentration (10% PEG-8000). **d** Turbidity assay was used to quantify phase separation of DBD truncated forms of PPARγ-FL (10 μmol/L protein, 150 μmol/L NaCl, 10% PEG-8000). OD600 was normalized to the measurement of mEGFP control (*n* = 3). Data were shown as means ± SEM. Top, a schematic diagram showing PPARγ with DBD truncated (top) and recombinant mEGFP fusion protein used here (bottom). **e** Representative fluorescence microscopy images for condensate formation of PPARγ truncations. Cells were transfected with PPARγ truncations for 48 h and imaged, respectively. **f** The disruption of zinc finger motif impaired the PPARγ phase separation. Representative images of wild-type PPARγ or PPARγ with the disruption of zinc finger motif (PPARγ C>A) fused to mEGFP in droplet formation assay (5 μmol/L protein, 150 μmol/L NaCl, 10% PEG-8000). All cysteines in zinc finger motif were mutated to alanines. **g** Column scatter charts display average droplet area of each image related to Fig. 2f. Data are shown as means ± SEM (*n* = 10). **h** Representative fluorescence microscopy images of a mixture of mEGFP-PPARγ-DBD/mCherry, mEGFP/mCherry-RXRα, mEGFP-PPARγ-DBD/mCherry-RXRα, and mEGFP-PPARγ-LBD/mCherry-RXRα respectively (2 μmol/L protein, 150 μmol/L NaCl, 10% PEG-8000). Scale bar, 5 μm. **i** Column scatter charts display the droplet diameter in reactions related to panel **h**. Data are shown as means ± SEM (*n* = 500). **j** Phase diagram of PPARγ-DBD in the presence of different concentrations of RXRα (150 μmol/L NaCl, 10% PEG-8000). One-way analysis of variance (ANOVA) for panels **a**, **d,** and **i**. Two-tailed unpaired *t*-test for panel **g**. *P < 0.05, **P < 0.01, ***P < 0.001, ****P < 0.0001. ns, not significant.

The structure of transcription factors usually consists of DBD and activation domain. Some transcription factors regulate gene activation through the phase-separating capacity of their activation domain-containing IDR. To identify the domains in PPARγ that are essential for phase separation, PPARγ-IDR (Supplementary Fig. S3a), PPARγ-NTD, PPARγ-DBD, and PPARγ-LBD were fused with mEGFP and purified (Supplementary Fig. S3b). PPARγ-DBD but not PPARγ-IDR, PPARγ-NTD and PPARγ-LBD produced a turbid solution in 10% PEG-8000 (Fig. 2a; Supplementary Fig. S3b). Furthermore, PPARγ-DBD formed micron-sized spherical droplets that were sensitive to changes in protein and salt concentrations (Fig. 2b, c; Supplementary Fig. S3c), similar to the characteristic of the full-length protein. Next, we performed semidenaturing detergent agarose gel electrophoresis (SDD-AGE) analysis (which detects protein aggregates) to address the role of DBD in PPARγ phase separation. The results showed that PPARγ has a high tendency to get aggregation, and DBD is a necessary domain for PPARγ aggregation (Supplementary Fig. S3d, e). To confirm that PPARγ aggregation further triggers phase separation occurrence, we carried out sedimentation assay to separate the condensed phase and the aqueous phase, followed by immunoblotting assays. Consistent with the results of SDD-AGE assay, PPARγ and PPARγ-DBD quantitatively entered the pellet fraction, but PPARγ-NTD, PPARγ-LBD and PPARγ-IDR remained mainly in the supernatant (Supplementary Fig. S3f). Moreover, PPARγ lacking the DBD failed to undergo phase separation (Fig. 2d; Supplementary Fig. S3g). Next, we investigated the role of DBD in condensate formation of PPARγ in cells. We constructed and expressed PPARγ truncations including PPARγ-DBD, PPARγ-LBD, PPARγ-NTD, and PPARγ-IDR in 3T3-L1 cells. Consistent with the results of phase separation in vitro, PPARγ-DBD but not PPARγ-IDR, PPARγ-NTD or PPARγ-LBD formed nuclear puncta in cells (Fig. 2e). Moreover, deletion of the DBD abolished the ability to form nuclear puncta (Fig. 2e), suggesting DBD is necessary for PPARγ phase separation in vitro and in cells.

The DBD of PPARγ contains two C4-type zinc fingers, each of which contains a group of four cysteine residues to form its primary and tertiary structure. The zinc finger motif has been reported to facilitate the occurrence of phase separation^24,26–29^. To explore the role of zinc finger motif in PPARγ phase separation, we replaced all the cysteines in zinc finger motif with alanines (Supplementary Fig. S4a). The disruption of zinc finger motif significantly impaired but not completely abolished the capability of PPARγ phase separation in vitro and condensate formation in cells (Fig. 2f, g; Supplementary Fig. S4b, c), suggesting zinc finger structure in the DBD contributed to its phase separation.

PPARγ activation is regulated by intramolecular interaction between various domain^30^. To further explore whether IDR, NTD, or LBD coordinates DBD phase separation through intramolecular interaction, PPARγ-DBD-LBD, PPARγ-NTD-DBD and PPARγ-IDR-NTD-DBD were fused with mEGFP and purified (Supplementary Fig. S5a). LBD and NTD enlarged the size of PPARγ-DBD droplets, whereas PPARγ-IDR functions as an inhibitory element (Supplementary Fig. S5b, c). These results strongly implicated intramolecular interaction between various domains that regulate PPARγ phase separation.

PPARγ interacts with RXRα through various types of domain–domain interactions. Next, we investigated whether the DBD is responsible for the co-phase separation of PPARγ and RXRα. The PPARγ-DBD phase separated with RXRα to form heterotypic droplets, which was not observed with the PPARγ-LBD (Fig. 2h). Moreover, the size of PPARγ-DBD/RXRα droplets was much larger than that of droplets formed by each protein alone, supporting the idea that PPARγ/RXRα heterodimerization promotes phase separation (Fig. 2h, i). As expected, the addition of RXRα protein also promoted PPARγ-DBD phase separation in a concentration-dependent manner (Fig. 2j; Supplementary Fig. S6). Thus, the DBD is necessary for PPARγ phase-separating with RXRα.

### PPARγ/RXRα condensates prefer to be enriched at PPREs to control gene expression

**Fig. 3:**
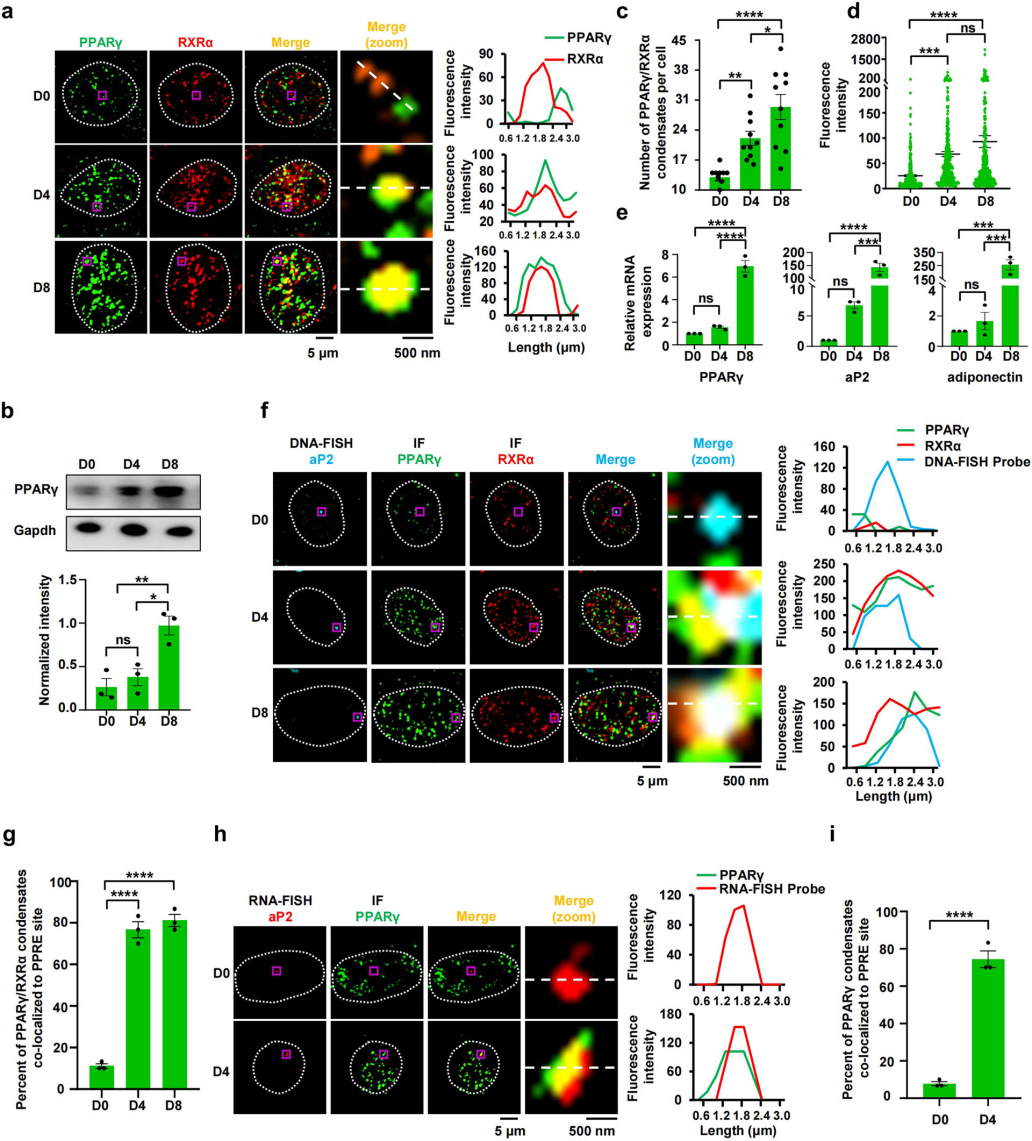
PPARγ/RXRα heterodimer condensates prefer to enrich at PPRE sites to promote target gene expression. **a** Co-immunostaining of PPARγ (green) and RXRα (red) in 3T3-L1 cells before and after differentiation (*n* = 10). Pre-adipocyte (undifferentiated) 3T3-L1 cells were grown to confluence (~48 h) and induced by IBMX, insulin and dexamethasone (day 0). The cells were induced to differentiate by changing to the media containing insulin (day 2) and then media were changed every two days. The differentiated adipocyte cells were fixed at day 0 (D 0), day 4 (D 4), and day 8 (D 8) and then immunofluorescence staining was performed. The white line displays nuclear periphery, determined by DAPI staining (not shown). Scale bar, 5 μm. The fourth column (merge (zoom)) displays a magnification of the purple box region in the third column for greater detail. Scale bar, 500 nm. Right panel, a line plot corresponding to magnified image. **b** Western blot analysis of PPARγ levels in 3T3-L1 cells before and after differentiation (*n* = 3). **c** The number of PPARγ/RXRα heterodimer condensates in 3T3-L1 cells before and after differentiation (*n* = 10). Data are shown as means ± SEM. **d** Fluorescence intensities of PPARγ/RXRα heterodimer condensates in 3T3-L1 cells before and after differentiation (*n* = 300). Data are shown as means ± SEM. **e** RT-qPCR analysis of PPARγ, aP2 and adiponectin transcription levels in 3T3-L1 cells before and after differentiation (*n* = 3). **f** Colocalization between PPARγ/RXRα heterodimer puncta and aP2-PPRE locus by IF and DNA-FISH in fixed 3T3-L1 cells before and after differentiation (*n* = 50). Scale bar, 5 μm. The fifth column (merge (zoom)) displays the magnification of the purple box region in the fourth column for greater detail. Scale bar, 500 nm. Right panel, the line plot corresponding to magnified image. **g** Quantification of DNA-FISH analysis using percentage of cells with PPARγ/RXRα condensates and aP2-PPRE locus colocalization in fixed 3T3-L1 cells before and after differentiation (*n* = 3). Data are shown as means ± SEM. **h** Colocalization between PPARγ/RXRα heterodimer puncta and aP2-PPRE locus by IF and RNA-FISH in fixed 3T3-L1 cells before and after differentiation (*n* = 50). **i** Quantification of RNA-FISH analysis using percentage of cells with PPARγ/RXRα condensates and aP2-PPRE locus colocalization in fixed 3T3-L1 cells before and after differentiation (*n* = 3). Data are shown as means ± SEM. One-way analysis of variance (ANOVA) for panels **b**, **c**, **d**, **e,** and **g**. Two-tailed unpaired *t*-test for panel **i**. *P < 0.05, **P < 0.01, ***P < 0.001, ****P < 0.0001. ns, not significant.

Phase separation is an important mechanism that enables transcription factors to achieve transcription activation in an efficient and specific manner^12,13,31^. To further examine whether PPARγ regulates transcription activation by condensate formation, we used a well-defined adipocyte differentiation model. Specifically, when 3T3-L1 cells were exposed to adipogenic inducers, PPARγ heterodimerized with RXRα to activate target gene expression, which initiated the differentiation of 3T3-L1 cells into adipocytes^32–35^. PPARγ-driven target gene transcription is also necessary for maintaining the differentiated state of mature adipocytes^34^. In 3T3-L1 cells, we observed only small and few heterotypic PPARγ or RXRα puncta in the nucleus (Fig. 3a). Importantly, during differentiation, when the expression of PPARγ was promoted, the number and fluorescence intensity of the heterotypic PPARγ and RXRα puncta progressively increased (Fig. 3a–d), thereby facilitating target gene expression (Fig. 3e). To further explore the causation, we treated 3T3-L1 cells with 2% 1,6-hexanediol, and found marked depletion of PPARγ puncta in cells with or without treatment of rosiglitazone (PPARγ agonist) associated with repression of PPARγ target gene expression (Supplementary Fig. S7). Thus, PPARγ/RXRα condensation is functionally relevant for PPARγ-driven transcription.

The PPARγ/RXRα transcriptional complex binds to PPREs to regulate target gene expression^1^. To determine whether PPARγ/RXRα condensates preferentially occur at PPREs in differentiated 3T3-L1 cells, we visualized PPARγ and RXRα with immunofluorescence and PPRE of gene encoding fatty acid-binding protein 4 (FABP4, also known as aP2), lipoprotein lipase (LPL) or the promoter region of gene encoding microtubule-associated protein tau (MAPT, as a control genomic region) with DNA-fluorescence in situ hybridization (FISH). In undifferentiated 3T3-L1 cells, PPARγ, RXRα, and the PPRE sites were separate (Fig. 3f, g; Supplementary Fig. S8a–d). In contrast, some PPARγ/RXRα heterotypic puncta emerged and specifically overlapped with the PPRE sites in differentiated 3T3-L1 cells, but they failed to localize at the genome site of MAPT promoter and activate MAPT gene expression (Fig. 3f, g; Supplementary Fig. S8). Similar tripartite colocalization was observed when 3T3-L1 cells were treated with rosiglitazone (Supplementary Fig. S9). As expected, PPARγ also colocalized with nascent RNA at the PPRE sites in differentiated 3T3-L1 cells (Fig. 3h, i). To test whether PPARγ/RXRα heterodimer specifically binds to PPRE to form phase-separated droplets, we incubated PPARγ/RXRα proteins with Cy5-labeled DNA. Upon mixing, PPRE, not control DNA, partitioned into PPARγ droplets to fuse into larger ones (Supplementary Fig. S10a, b). Similar results were obtained in the reaction of PPARγ with DNA (Supplementary Fig. S10c, d). However, the addition of DNA containing PPRE region failed to trigger PPARγ/RXRα complex phase separation, suggesting that PPRE is not a driver for PPARγ/RXRα heterodimer phase separation, but partitions into PPARγ/RXRα heterodimer droplets to form heterotypic coacervates (Supplementary Fig. S10e, f). Furthermore, DNA addition was not able to change the inner mobility of PPARγ/RXRα condensation, suggesting that the droplets of PPARγ/RXRα/DNA complex represent gel or solid-like state (Supplementary Fig. S10g, h).

These data suggest that PPARγ/RXRα heterodimer condensates prefer to be enriched at PPREs, which then promote transcription of the target genes. The zinc finger region in PPARγ is responsible for binding to PPRE^30^. PPARγ with the disruption of zinc finger motif in DBD impaired the ability to form condensation in the nucleus, and lost the capacity to be enriched in PPRE sites (Supplementary Fig. S11). Furthermore, PPARγ/RXRα condensates did not appear at the aP2-PPRE with a 5′ half-site mutation (A>G) or a 3′ half-site mutation (T>C) (Supplementary Fig. S12a–d), but did localize at the wild-type aP2-PPRE site. aP2-PPRE with mutation failed to fuse into PPARγ/RXRα droplets and blocked rosiglitazone-induced aP2 mRNA expression without disrupting the interaction between PPRE and PPARγ (Supplementary Fig. S12e–h), suggesting that the assembly of PPARγ/RXRα complex at specific PPREs through phase separation is crucial for efficient and PPARγ-specific transcriptional activation.

### Enforced formation of PPARγ/RXRα condensates preferentially occurs at PPRE sites and then significantly promotes the target gene expression

**Fig. 4:**
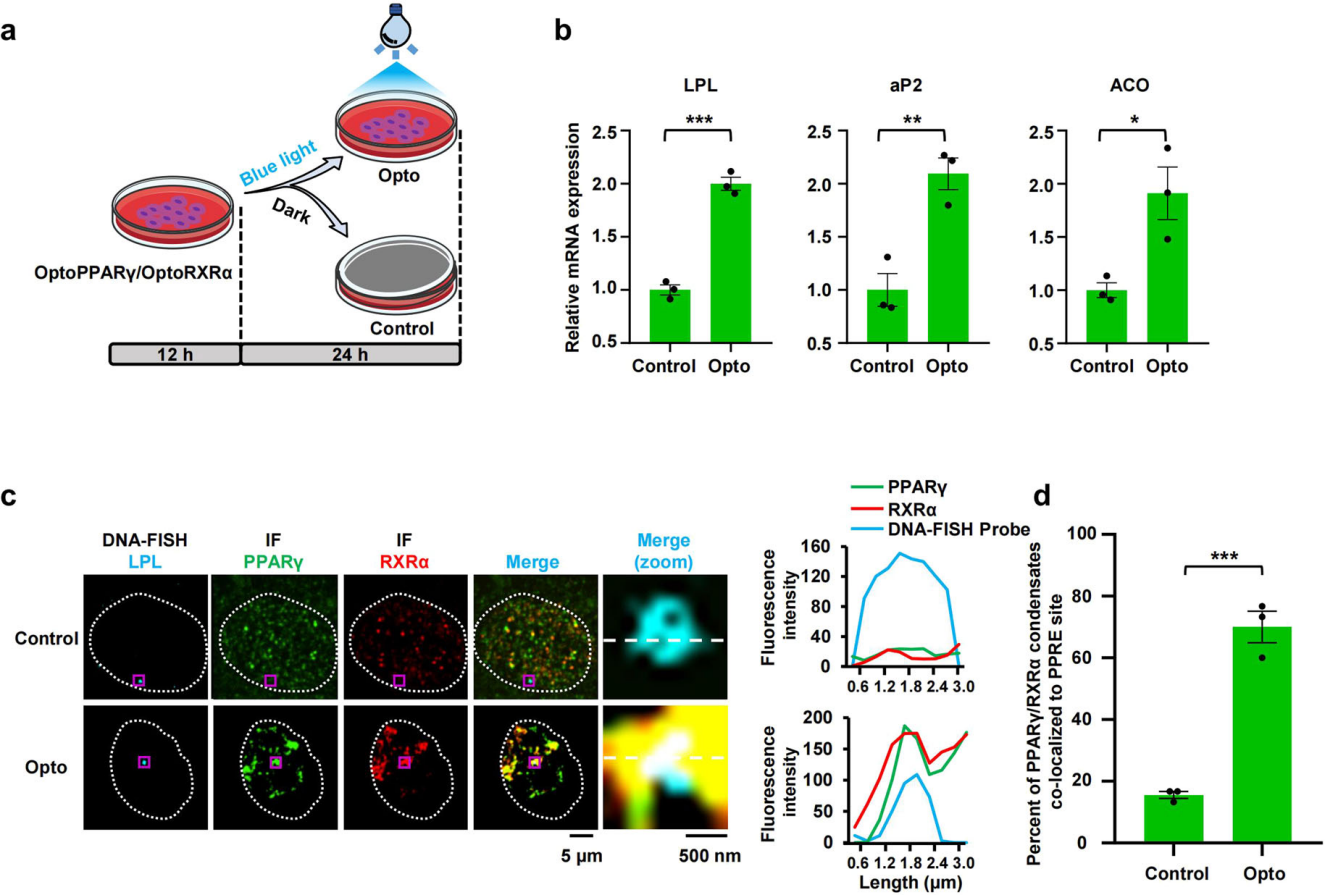
Enforced formation of PPARγ/RXRα condensates remarkably generate specific downstream outcomes. **a** Schematic timeline of seeding and blue light illumination of pre-3T3-L1 cells. 3T3-L1 cells with stable expression of optoPPARγ and optoRXRα were seeded in the dish for 12 h and then treated by blue light for 24 h. Dark-treated cells were referred as Control, and blue light-treated cells were referred as Opto. **b** RT-qPCR analysis of LPL, aP2, and ACO transcription levels in dark-treated cells and light-treated cells. Data are shown as means ± SEM (*n* = 3). **c** Colocalization between optoPPARγ/optoRXRα heterodimer puncta and LPL-PPRE locus by IF and DNA-FISH in fixed Control-3T3-L1 cell and Opto-3T3-L1 cell (*n* = 50). Separate images of the indicated LPL-PPRE probe (first column), PPARγ (second column) and RXRα (third column) are shown, accompanied with an image showing the merged channels (the fourth column, overlapping signal in white). Scale bar, 5 μm. The fifth column (merge (zoom)) displays the magnification of the purple box region in the fourth column for greater detail. Scale bar, 500 nm. Right panel, the line plot corresponding to magnified image. **d** Quantification of DNA-FISH analysis using percentage of cells with PPARγ/RXRα condensates and LPL-PPRE locus colocalization in fixed Control- and Opto-3T3-L1 cell (*n* = 3). Data are shown as means ± SEM. Statistics: two-tailed unpaired *t*-test. *P < 0.05, **P < 0.01, ***P < 0.001, ****P < 0.0001. ns, not significant.

To gain further insights into the function of PPRE-dependent PPARγ/RXRα condensation in transcriptional activation, we performed optogenetic droplet assays to demonstrate the importance of PPARγ/RXRα condensation for the activation of target genes based on light-dependent oligomerization of cryptochrome 2 protein (Cry2)^36,37^. A growing body of studies involving light-activated condensation has revealed the function of phase separation in reorganizing the genome for transcription and indicated that nuclear condensates can be mechanically pulled together to generate specific downstream outcomes^38,39^. To this end, we stably expressed PPARγ-mCherry-Cry2olig and/or RXRα-mEGFP-Cry2olig fusion proteins (optoPPARγ and optoRXRα) in 3T3-L1 cells and HEK293T cells (Fig. 4a). When the fusion proteins were separately expressed, they formed only small droplets that fused into larger droplets following blue light illumination (Supplementary Fig. S13a, b); when coexpressed, the optoPPARγ and optoRXRα droplets coalesced (Supplementary Fig. S13c, d). With these findings, we evaluated the roles of optoPPARγ/optoRXRα heterotypic droplets in target gene expression and found that 24 h of illumination markedly enhanced the expression of PPARγ-responsive genes in both 3T3-L1 and HEK293T cells (Fig. 4b; Supplementary Fig. S14a), and this effect was associated with the colocalization of PPARγ/RXRα heterotypic droplets with PPRE sites (Fig. 4c, d; Supplementary Fig. S14b, c). These results indicate that enforced condensation of the PPARγ/RXRα heterodimer by phase separation was sufficient to increase PPARγ-targeted gene transcription.

## Discussion

PPARγ heterodimerizes with RXRα to activate the expression of various PPARγ target genes in response to lipid and glucose metabolism^1,5^. How PPARγ orchestrates the transcriptional response to generate specific downstream outcomes is unclear. Here, we demonstrate that the transcription factor PPARγ phase separates with its heterodimer partner RXRα to selectively form nuclear condensates at PPREs to efficiently activate transcription. Our results identified features of DNA sequences that can assemble transcription condensates of PPARγ at specific genomic loci through phase separation and uncovered a novel framework to account for the PPRE-mediated transcriptional output of PPARγ.

The DBD in transcription factor functions as an anchor and selectively binds specific super-enhancers or promoters to initiate or enhance transcription^12,40,41^. The activation domains in which IDRs are enriched mediate the assembly of many transcription proteins through IDR-mediated phase separation to control gene activation^42,43^. In our study, we found that phase separation of PPARγ is mediated by its DBD instead of IDR or NTD (activation domain). The DBD of PPARγ or RXRα contains two highly conserved zinc fingers, with the P-box region in zinc finger I^30^. Recently, the zinc finger motif has been reported to have a similar function as IDR, which is capable of enhancing protein oligomerization and contributing to the occurrence of phase separation^26,27,44^. PPARγ with the disruption of zinc finger motif in DBD showed remarkably attenuated capability of phase separation in vitro and in cells. In addition, the distribution of opposite charges often acts as a driver for phase separation^45^. DBD of PPARγ contains distributed basic and acidic residues, leading to a strong self-assembling tendency for phase separation^45,46^. Therefore, DBD characterized by zinc finger motif and distribution of charged residues is a crucial domain for PPARγ phase separation. PPAR isoforms include PPARα, PPARβ, and PPARγ that share high homologous sequence in DBDs^1^. Due to sequence similarity and zinc fingers structure of DBD, PPARα, and PPARβ are able to phase separate in vitro and in cells^5^, suggesting that the transcription factors sharing similar motif of zinc fingers might phase separate.

PPARγ–RXRα phase separation is dependent on residues of DBD. Previous structural analysis suggests that PPARγ interacts with RXRα through many types of domain–domain interactions. Among them, RXRα-DBD (the short carboxy-terminal extension) is able to form dimers with PPARγ-DBD, and this interaction enables the complex to be more flexible, which may facilitate the occurrence of phase separation^30,47–49^. Deletion of PPARγ-DBD significantly impaired not only PPARγ phase separation but also PPARγ phase separation with RXRα. Here, we demonstrated the role of DBD in PPARγ oligomerization and its additional multivalent interactions with RXRα.

N-terminal IDRs in transcription factors with low amino acid sequence complexity often act as drivers for phase separation by regulating oligomerization or facilitating multivalent interactions^16,50^. However, domain with different biochemical nature in transcription factors presents different phase separation potential. The N-terminal IDR is essential for the formation of estrogen receptor (ER) condensates whereas LBD is required for the formation of glucocorticoid receptor (GR) foci^12,51^. Interestingly, the DBD of androgen receptor (AR) can bind RNA and undergoes RNA-dependent phase separation. Furthermore, the long N-terminal disordered transactivation domain is able to inhibit the phase separation of AR mutants without LBD^52^. We here found DBD is crucial for PPARγ phase separation in vitro and in cells, and N-terminal IDR functions as an inhibitory domain for phase separation of DBD, whereas that LBD acts as a contributory region. Thus, PPARγ phase separation is regulated by intramolecular interaction.

Adipogenesis is a key process that determines the size and total number of mature adipocytes. PPARγ is undoubtedly the most important transcription factor that regulates adipocyte differentiation by binding various PPREs within promoters of adipogenesis-related genes^6,32,33^. Recent studies have revealed that phase-separated transcription factors form biomolecular condensates that concentrate the transcription machinery at specific loci to regulate transcription^13,15,53–56^. Here, we clarified the role of PPREs in the assembly of the PPARγ transcription machinery. PPARγ–RXRα complexes specifically recruited PPRE to form heterotic droplets of the PPARγ–RXRα–DNA complex, suggesting that PPREs are involved in the formation of transcription machinery clustering through multivalent protein–DNA interactions. In living cells, PPARγ–RXRα heterodimers are enriched at PPREs to form biomolecular condensates, suggesting that specific types of motif compositions of DNA drive localized formation of PPARγ transcriptional condensates. DNA-involved formation of biomolecular condensates plays distinct roles in the regulation of transcriptional activity. Human heterochromatin protein 1α and vernalization 1 induce transcriptional repression or gene silencing due to liquid-liquid phase separation-mediated DNA compaction. Alternatively, genomic DNA functions as a scaffold for the formation of biomolecular condensates at specific loci to promote gene transcription^13,53,56,57^. For PPARγ, PPRE-specific phase separation of PPARγ–RXRα heterodimers promotes the expression of its target genes. Furthermore, the mutant PPRE failed to drive the localized formation of PPARγ transcriptional condensates at PPREs and thereby impaired transcriptional activity. Thus, PPRE-specific phase separation of PPARγ–RXRα heterodimers controls gene transcription.

Proteins assembly and condensation regulates distinct cellular functions via liquid, gel or/and solid-like phase separation^58^. The physical output of a homogeneous phase separation is often not enough to reflect the full complexity of intracellular condensates^59^. Moreover, the liquid-like condensate is in a metastable state, and some liquid-like compartments can turn into more stable structures and finally gel/solid-like aggregates over time. These processes are contributed by an increase in the interaction entanglement of key components^60–64^. The droplets of PPARγ alone showed moderate mobility, whereas PPARγ failed to recovery when heterodimerized with RXRα in vitro and in cells, indicating that the solid-like characteristics of PPARγ–RXRα transcriptional complex may act as an underlying mechanism for stable expression of the target gene.

The function of phase separation in reorganizing the genome for transcription has been well studied using biophysically relevant approaches, including light-induced phase-separation systems^12,13,38,65,66^. In our study, we found that PPARγ–RXRα heterodimer condensates selectively accumulated at PPRE loci. In addition, this phase separation-mediated genome reorganization specifically enhanced the expression of PPARγ target genes, which is consistent with a proposed characteristic of nuclear condensates that they preferentially pull in the targeted genomic loci to regulate transcription outcome^12,13,15,39^. The possible reasons are that these condensates increase the effective local concentration of proteins needed for transcriptional activation and frequency of components interaction to promote transcription stability. However, the extent to which phase separation is necessary for transcriptional activation still need to be studied in the future.

In summary, our findings show that the transcription factor PPARγ phase separates with its heterodimer partner RXRα to concentrate the transcription machinery specifically at PPREs to efficiently regulate the transcription of PPARγ-targeted genes. This study provides an alternative strategy for the modification of PPARγ target gene expression through phase separation, which may alter the course of obesity and insulin resistance that involve PPARγ signaling.

## Materials and methods

### Plasmid construction

DNA fragments encoding the proteins of interest were synthesized by GenScript Biotech Corporation (Nanjing, China) and amplified by PCR with Phanta® Max Super-Fidelity DNA Polymerase (Vazyme, P505-d1), while the coding region for PPARγ truncations (residues 1–39, residues 31–108, residues 138–221, residues 1–221, residues 31–221, residues 138–504 and residues 237–504) were generated by PCR from a plasmid containing full-length *PPARγ* with appropriate sets of primers. Exnase (Vazyme, C214-02-AF) was used to insert these sequences into the pET-28a vector containing an mCherry or mEGFP tag. Plasmid inserts were confirmed by BioSune Sanger sequencing, reading from both ends of the insert. For the construction of sgRNA expression plasmids, oligos were custom synthesized, annealed and cloned into pGL3-U6-sgRNA-EGFP vector (Addgene, 107721).

### Cell culture

HEK293T cells and 3T3-L1 cells were obtained from American type culture collection (ATCC, https://www.atcc.org/). Cells were maintained in Dulbecco modified Eagle medium (DMEM; Gibco, C11995500BT) supplemented with 10% (v/v) fetal bovine serum (Gemini, 900108) and 1% (v/v) penicillin/streptomycin (Gibco, 15140122) under standard tissue-culture conditions (37 °C, 5% CO_2_).

### Construction of Cell with PPRE mutation

To construct mutant cell lines, 3T3-L1 cells were seeded in a 24-well plate and transfected with 500 ng sgRNA and 1000 ng base editor plasmid (pCMV_ABEmax (Addgene, 112095) or pCMV_AncBE4max (Addgene, 112094)) using Lipofectamine 3000 transfection kit (Invitrogen, L3000015). After transfection for 6 h, the medium was changed to fresh DMEM supplemented with 10% (v/v) fetal bovine serum. After cells were cultured for 72 h, the GFP-positive cells were harvested from fluorescence-activated cell sorting (FACS). The genomic DNA of GFP-positive cells was extracted using QuickExtract™ DNA Extraction Solution (Lucigen, QE09050), the targeting sequence was amplified by PCR and analyzed by BioSune Sanger sequencing. The sgRNAs used are listed in Supplementary Table S1.

### 3T3-L1 cells differentiation

3T3-L1 cells were grown in 24-well plates to full confluence for 2 days and then differentiation medium (DM) containing 10 μg/mL insulin (Sigma, I-9278), 0.5 μmol/L dexamethasone (Sigma, D-4902), and 0.8 mmol/L isobutylmethyl xanthine (IBMX; Sigma, I-7018) was added to the culture (Day 0). After 2 days, the medium was changed to complete DMEM with 10 μg/mL insulin (Day 2). Then medium was changed to complete DMEM every two days. Full differentiation is usually achieved on Day 8.

### Rosiglitazone treatment

Rosiglitazone (ENZO, ALX-350-125-M025) was dissolved in DMSO and was added to the medium at indicated concentrations. DMSO was added to the cells as the untreated control.

### Lentivirus production and transduction

Lentiviral transfer constructs encoding PPARγ-mCherry, PPARγ-mCherry-Cry2, RXRα-mEGFP, RXRα-mEGFP-Cry2, mCherry-Cry2 or mEGFP-Cry2 fragments were transfected with packaging plasmids into HEK293T cells using Lipofectamine 3000 transfection kit (Invitrogen, L3000015) according to the manufacturer’s instructions. Lentiviral supernatants were collected after transfection for 48 h or 72 h and centrifuged at 27,000 rpm for 2 h at 4 °C. The pellets were then dissolved with DMEM and stored at –80 °C. HEK293T or 3T3-L1 cells were infected by adding filtered viral supernatant mixed with 6 μg/mL polybrene (Yeason, 40804ES76). Media changes were performed after infection for 48 h and cells that stably expressing optoPPARγ/optoRXRα were constructed via FACS according to the fluorescence tag.

### Protein disorder prediction

The prediction of protein IDRs for PPARγ was performed using the PONDR@ webtool by VSL2 algorithm (http://www.pondr.com/).

### Protein expression and purification

For protein expression, the recombinant plasmids with DNA fragments encoding the proteins of interest were transformed into chemically competent *E. coli* BL21 (DE3) (Trans, CD601) under the selection of kanamycin. A fresh bacterial colony was selected and grown in LB medium at 37 °C until OD_600_ attained 0.7. Cells were then induced with 1 mmol/L IPTG (Diamond, 367-93-1) and cultured at 16 °C for 20 h. For the following proteins: mEGFP-PPARβ, mEGFP-PPARγ, mEGFP-PPARγ-NTD, mEGFP-PPARγ-IDR, mEGFP-PPARγ-LBD, mEGFP-PPARγ-DBD, mCherry-RXRα, mEGFP-PPARγ^∆DBD^, mEGFP-PPARγ-IDR-NTD-DBD, mEGFP-PPARγ (C>A) mutant, mEGFP and mCherry, cells were harvested by centrifugation and resuspended in 15 mL of buffer A (20 mmol/L Tris, pH 7.5, 500 mmol/L NaCl, 10% (v/v) glycerol, 1 mmol/L phenylmethanesulfonyl fluoride (PMSF)), and for mEGFP-PPARα, mEGFP-PPARγ-DBD-LBD, mEGFP-PPARγ-NTD-DBD protein purification, harvested cells were resuspended in 15 mL of buffer B (20 mmol/L Tris, pH 7.0, 500 mmol/L NaCl, 10% (v/v) glycerol, 1 mmol/L PMSF). After cells lysed by EmulsiFlex-C3 (Avestin, Ottawa, Canada), the lysates were cleared by centrifuging at 35,000 rpm for 60 min and then the supernatants were collected. The supernatant was loaded on a polypropylene column (QIAGEN, 34964) containing 4 mL pre-equilibrated His60-Ni-Superflow-Resin (TaKaRa, 635660). Proteins were finally eluted by 10 mL buffer A or buffer B containing 500 mmol/L imidazole. After that, proteins were further purified by size exclusion with a Superdex-200 column on an AEKTA purifier (GE Healthcare Life Sciences, Boston, USA). Then, proteins were concentrated to 2 mL volume using Amicon Ultra centrifugal filters (Millipore, UFC901096), the protein concentration was determined using BCA protein assay kit (Thermo Fisher scientific, NCI3227CH) according to the manufacturer’s instructions. Finally, the purified proteins were snap frozen as 200 μL aliquots in tubes in liquid nitrogen and stored at –80 °C.

### In vitro droplet assay

All purified mEGFP or mCherry fusion proteins were concentrated to the same volume (50 μL) with the same concentration (5 μmol/L), and 10% (v/v) PEG-8000 as crowding agent was added immediately. Then, each protein solution was transferred to a highly transparent 1.5 mL tube which was fixed to a homemade shelf. The salt-dependent phase separation was performed by making the protein solution of the protein of interest with specified concentration of NaCl. At each specific concentration of Na^+^, phase-separation reactions were prepared at different protein concentrations. Reactions were then transferred in 96-well glass bottom plate (Cellvis, P96-1.5H-N) and observed under a Nikon Spinning Disk microscope equipped with 100× oil immersion objective. For droplet assay for concentration-dependent phase separation, these proteins formed droplet with the indicated concentration in 96-well glass bottom plate (Cellvis, P96-1.5H-N). Droplets were also visualized with Nikon Spinning Disk microscope.

### Sedimentation assay

For the sedimentation assay, samples were centrifuged at 20,000× *g* for 15 min in a tube at 4 °C. After centrifugation, the resulting supernatants were immediately transferred into new tubes and the remaining pellet fractions were washed once and suspended using buffer A with equal volume to the supernatants. Next, 10 μL of 10-fold diluted supernatant and pellet samples were reduced with 1 mmol/L DTT and loaded into sodium dodecyl sulfate polyacrylamide gel electrophoresis (SDS-PAGE) for western blotting analysis. Anti-GFP primary antibodies (ABvlonal, AE078) and horse radish peroxidase-conjugated secondary antibodies were used for protein detection. The immunoblotting signals were visualized by Immobilon Western enhanced chemiluminescent solution (Millipore, WBKLS0100).

### SDD-AGE assay

The SDD-AGE assay was performed according to the protocols described previously^67^. Briefly, 2 μg indicated proteins were diluted to 10 μL and loaded with loading buffer (EpiZyme, LT101). Newly prepared 1.5% (v/v) agaroses gel with 0.1% (v/v) SDS was pre-run by electrophoresis for 1 h with a constant voltage of 100 V at 4 °C, followed by samples loading and running for another 1 h under the same running condition. Finally, the proteins were transferred to PVDF membrane (Millipore, IPVH00010) for western blot assay with 6× His mAb/HRP conjugate (TaKaRa, 631210).

### FRAP analysis

The experiment was performed using Nikon Spinning Disk microscope equipped with two laser systems. A region of the indicated protein droplets was bleached by a 488 nm or 561 nm wavelength laser with the light intensity of 80%. Only the center of the droplets was bleached. Fluorescence intensity recovery data were recorded. Fluorescence intensity was obtained using FIJI (National Institutes of Health, Bethesda, USA). Fluorescence intensities of the region of interest (ROI) was subtracted by background intensity and then normalized by pre-bleached intensities of the ROIs. The FRAP recovery curve was fit to the formula described previously^68^.

### Construction of blue light laser source

For our experiments, we made a 6 × 6 compact form laser diode array (Sharp, model NO. GH04580A2G) with 450 nm emitting wavelength. All laser diodes were soldered on the PCB board with 1 cm interval and powered via a voltage regulator chip. A digital potentiometer was connected in series with a 3 kΩ resistor (*R*_1_) in order to adjust the output voltage (*V*_out_) of the voltage regulator chip. We chose NodeMCU 1.0 to adjust the resistance of the digital potentiometer (*R*_dp_) and the pulse time of the output voltage. According to the product manual, *V*_out_ can be represented as ${{{{V}}}}_{{{{\mathrm{out}}}}} = 1.216 \times (1 + R_{{{{\mathrm{dp}}}}}/R_1)$. For portability, the whole blue light laser source can be powered via either a power adapter (2000 mA, 5 V) or 3× rechargeable battery (1.5 V). We also designed an aluminum alloy shelf that can be used to adjust the distance between the light source and illuminated target, the blue light laser source was held 5.5 cm below the shelf. The code for controlling is attached below.

Arduino Code:

void setup() {

pinMode(POWER_PIN, OUTPUT);

digitalWrite(POWER_PIN, HIGH);

SPI.begin();

pinMode(SS, OUTPUT);

digitalWrite(SS, LOW);

SPI.transfer(VOUT);

digitalWrite(SS, HIGH);

}

void loop() {

digitalWrite(POWER_PIN, HIGH);

delay(ON_DELAY);

digitalWrite(POWER_PIN, LOW);

delay(OFF_DELAY);

}

### Blue light irradiation to cells

Cells stably expressing Cry2 fusion proteins were stimulated by blue light laser source and cultured in an incubator. *V*_out_ was set to 3.3 V and maintained for 20 s with 5 s interval. For RNA collection, cells were exposed to blue light for 24 h. For immunofluorescence, cells were exposed to blue light for 4 h.

### Western blotting

The 3T3-L1 cells were rinsed with PBS (pH 7.4) and lysed in radio immunoprecipitation assay lysis buffer (Beyotime, P0013B) supplemented with a protease and phosphatase inhibitor cocktail (Thermo Fisher scientific, 78440) on ice for 30 min. Cell lysates were centrifuged for 20 min (12,000× *g*, 4 °C) and the protein concentration was measured using BCA protein assay kit (Thermo Fisher scientific, NCI3227CH). Equal amounts of protein (20 μg) were resolved by SDS-PAGE and transferred to polyvinylidene fluoride (PVDF) membranes (Millipore, IPVH00010). The membranes were blocked for 1 h at room temperature in Tris-buffered saline and 0.1% Tween 20 (TBST) containing 5% (w/v) nonfat milk and then incubated with primary antibodies at 4 °C overnight. The protein bands were detected with horse radish peroxidase-conjugated secondary antibodies and Immobilon Western enhanced chemiluminescent solution (Millipore, WBKLS0100). The protein levels were analyzed using Western blots with corresponding antibodies. The protein levels were normalized by probing the same blots with a GAPDH antibody.

### RNA extraction and real-time quantitative PCR (RT-qPCR)

Total RNAs from HEK293T and 3T3-L1 cells were isolated from cells by Trizol (TaKaRa, 9108). 1 μg RNA was reverse transcribed into cDNA using Reverse Transcriptase (Vazyme, R223-01-AB) according to the manufacturer’s instructions. Gene expression was assayed by real-time PCR using 2× ChamQ SYBR (Vazyme, Q331-AA) on ABI ViiA™ 7 real-time PCR system (Applied Biosystems, Carlsbad, USA). The mRNA levels of all genes were normalized using GAPDH or β-Actin as an internal control. Sequences for primers are listed in Supplementary Table S2. Measurements were performed in triplicate for each biological sample.

### Immunofluorescence

Cells were seeded to reach 40%–60% confluence in 96-well or 24-well glass bottom plates (Cellvis, Mountain View, USA) coated with poly-L-lysine (Sigma, P4707). After washed with PBS, cells were fixed in 4% paraformaldehyde for 15 min at room temperature and washed with PBS for three times. Next, fixed cells were incubated in blocking solution (containing 5% (v/v) Normal Goat Serum (Bioss Antibodies, C01-03001), 0.3% (v/v) Triton X-100 in PBS) for 2 h at room temperature. After that, the cells were incubated with primary antibody of RXRα (Santa cruz, sc-515929), PPARγ (Cell Signaling, 2435 S) or mCherry (Thermo Fisher scientific, M11217) overnight at 4 °C. Next, cells were washed three times in PBS and then incubated with secondary antibodies conjugated to Alexa Fluor 555 (Cell Signaling, 4409 S), Alexa Fluor 488 (Cell Signaling, 4412 S) or Alexa Fluor Plus 488 (Thermo Fisher scientific, A48262) at 1:1000 dilution for 2 h at room temperature. During this period, 96-well plate was wrapped in foil to keep it in dark environment. Cells were then washed three times in PBS for 10 min. Nuclei staining was performed with DAPI (YEASEN, 40728ES10). Images were acquired at the Nikon Spinning Disk microscope with 100× oil immersion objectives. Fluorescence intensity was obtained using FIJI (National Institutes of Health, Bethesda, USA).

### DNA-FISH

DNA-FISH was performed as described previously^69^. Briefly, cells were grown in 96-well or 24-well glass bottom plate. After immunofluorescence as described above, cells were incubated in 2 mol/L hydrochloric acid for 5 min and then washed three times in PBS. Next, cells were treated with 0.4 mg/mL RNaseA (TIANGEN, 03313) in PBS for 10 min at 37 °C. Cells were then incubated with 70% (v/v) formamide in saline sodium citrate (SSC) buffer (Sangon Biotech, B548109-0200) at 75 °C for 10 min. Cells were sequentially incubated in 70%, 85%, and 100% cold ethanol for 1 min at room temperature, respectively. After removing excess ethanol, pre-hybridization buffer (50% (v/v) formamide, 5× SSC, 9 mmol/L citric acid, pH 6.0, 0.1% (v/v) Tween-20, 50 μg/mL 1× heparin, 10% (v/v) dextran sulfate) was added. During this process, the temperature needs to be maintained at 45 °C for 30 min. Cells were then incubated with probe solution (pre-hybridization buffer with 0.01 μmol/L FISH probes) at 45 °C overnight. Washing solution (50% (v/v) formamide, 5× SSC, 9 mmol/L citric acid, pH 6.0, 0.1% (v/v) Tween-20, 50 μg/mL heparin) and SSCT solution (5× SSC, 0.1% (v/v) Tween-20) were prepared and pre-heated at 45 °C. After removing excess probe solution, cells were sequentially washed in 25%, 50%, and 75% SSCT diluted in washing solution at 45 °C, respectively. Cells were then incubated in 100% SSCT for 30 min at 45 °C. After washing step, amplification buffer (5× SSC, 0.1% (v/v) Tween-20, 10% (v/v) dextran sulfate) was added to perform the pre-amplification at room temperature for 30 min. Custom-designed DNA hairpins were annealed (95 °C for 90 s, 25 °C for 5 min, ramp rate at 3%). Cells were then incubated with DNA hairpin (0.06 μmol/L) in amplification buffer overnight. After removing the excess hairpin solution, we washed cells four times with 5× SSC. Nuclei staining was performed with DAPI. Sequences of DNA-FISH probes were listed in Supplementary Table S3. Hybridization chain reaction (HCR) amplifier sequences were from previous study^69^. Images were acquired at the Nikon Spinning Disk microscope with 100× oil immersion objective.

### RNA-FISH

Immunofluorescence was performed in a RNase-free environment according to described above. All pipettes and bench were treated with RNaseZap (Life Technologies, AM9780). Then, cells were fixed with 4% (v/v) PFA for 24 h at 4 °C and washed three times with RNase-free PBS. Permeabilization and dehydration of cells were performed using 100% (v/v) methanol for washing for 10 min and this step was repeated four times. Rehydration step was performed using a series of graded methanol/PBST (75% (v/v) methanol, 50% (v/v) methanol, 25% (v/v) methanol, 0% (v/v) methanol) for washing for 5 min, respectively. RNA probes were designed to hybridize the exon region of the transcripts of PPRE-associated gene. RNA probe hybridization step was similar to DNA-FISH assay described above but in RNase-free solutions. Sequences of RNA-FISH probes are listed in Supplementary Table S4. HCR amplifier sequences were from previous study^69^. Images were acquired at the Nikon Spinning Disk microscope with 100× oil immersion objective.

### Statistical analysis

All statistical analyses were done using OriginPro (2019b, OriginLab, Northampton, USA) or Microsoft Excel (Professional 2019, Microsoft Corporation, Redmond, USA). The outcomes of all statistical tests including number of samples and *P* values are revealed in the corresponding figure legends. Results were presented as means ± SEM. The significance of *P* values is represented as follows: *P < 0.05; **P < 0.01, ***P < 0.001, ****P < 0.0001.

## Supplementary information

Supplementary Information

## Data Availability

The data that support the findings of this study are available from the corresponding author upon reasonable request.
