# Supplementary material for: PPARγ phase separates with RXRα at PPREs to regulate target gene expression
Source: Cell Discov. 2022 Apr 26;8:37. doi: 10.1038/s41421-022-00388-0 (PMC9043196; doi:10.1038/s41421-022-00388-0)
Supplement: Supplementary file 1 — Supplementary Information [file 41421_2022_388_MOESM1_ESM.pdf]

Supplementary materials

PPARγ phase separates with RXRα at PPREs to regulate target gene expression

Zhean Li[1,2], Lingling Luo[3], Wenxia Yu[1], Ping Li[4], Danfeng Ou[5], Jia Liu[2], Hanhui Ma[1], Qinhu Sun[6], Aibin Liang[4], Cheng Huang[3], Tian Chi[1], Xingxu Huang[1,7], Yu Zhang[1]

Correspondence: Xingxu Huang (huangxx@shanghaitech.edu), Yu Zhang (zhangy@shanghaitech.edu.cn)

This file includes:
Supplementary Tables S1–S4
Supplementary Figs. S1–S14

Supplementary Table S1. sgRNAs used in this study.

sgRNA Name	sgRNA sequence
PPRE (5’ half site mutation A > G)	ACTGGATCAGAGTTCACTAG
PPRE (3’ half site mutation T > C)	ACTTGAGACTAGGTCATTCT
PPRE (3’ half site mutation G > A)	ACTTGAGACTAGGTCATTCT

Supplementary Table S2. Sequences of the primers used in real time PCR.

Gene	Forward primer	Reverse primer
GAPDH (m)	GGTGAAGGTCGGTGTGAACG	CTCGCTCCTGGAAGATGGTG
β-actin (m)	GTCCCTGACCCTCCCAAAAG	GCTGCCTCAACACCTCAACCC
ACC (m)	GAATCTCCTGGTGACAATGCTTATT	GGTCTTGCTGAGTTGGGTTAGCT
aP2 (m)	CATGGCCAAGCCCAACAT	CGCCCAGTTTGAAGGAAATC
ACO (m)	GCTGGCCGTGTCCATAGC	TTATCCGTGGGTCCAAACTGA
UCP2 (m)	GGGCACTGCAAGCATGTGTA	TCAGATTCCTGGGCAAGTCACT
adiponectin (m)	AAAGGGCTCAGGATGCTACTG	TGGGCAGGATTAAGAGGAACA
LPL (m)	ATCGGAGAACTGCTCATGATGA	CGGATCCTCTCGATGACGAA
MAPT (m)	GCTGTAGCAGTCCTGAAAGGTGAA	CTTCCTCCACTGTGGCTGTTTGTT
GAPDH (h)	AGAAGGCTGGGGCTCATTTG	AGGGGCCATCCACAGTCTTC
LPL (h)	TATGCAGAAGCCCCGAGT	ATGAAGAGATGAATGGAG
ACC (h)	GGATGGTGTTCACTCGGTAATAGA	GGGTGATATGTGCTGCGTCAT
ACO (h)	GGCATCGCTGACCCTGATGA	CGCAGTTGCCTGGTGAAGCA

Supplementary Table S3. Probes sequence for DNA-FISH.

Site	Probe sequence	Amplifier
aP2-PPRE (m)	ATGGATTTGCCTCATTGTGGAGGAGACAATTATCTTGGACACATTTGACC	DNA HCR B1
LPL-PPRE (m)	GCAGACGGAAAAATTTGCTTTGTTAGGCTCCTGTTCGCTTAGCTTAAGGG	DNA HCR B1
MAPT-promoter (m)	GCTGCAGAGAGAGGCGTGTGCAGAGCAGTTACTTAGCATTCAAGGGTGGA	DNA HCR B1
LPL-PPRE (h)	AAGAATGGCCACTGTGCCAGATGCCCCTGACCAGCGTTGCCCATTTGAAT	DNA HCR B1
ACC-PPRE (h)	AACGGTGCCAGCGAGAAGAAGGGGAAAGGGCAGGAAGGAAAGCTTGCTTG	DNA HCR B1

Supplementary Table S4. Probes sequence for RNA-FISH.

Site	Probe sequence	Amplifier
aP2-exon1 (m)	CGAAGTTTTCACTGGAGACAAGCTTCCAGGTTCCCACAAAGGCATCACAC	DNA HCR B1
aP2-exon1 (m)	CATGCCTGCCACTTTCCTTGTGGCAAAGCCCACTCCCACTTCTTTCATGT	DNA HCR B1

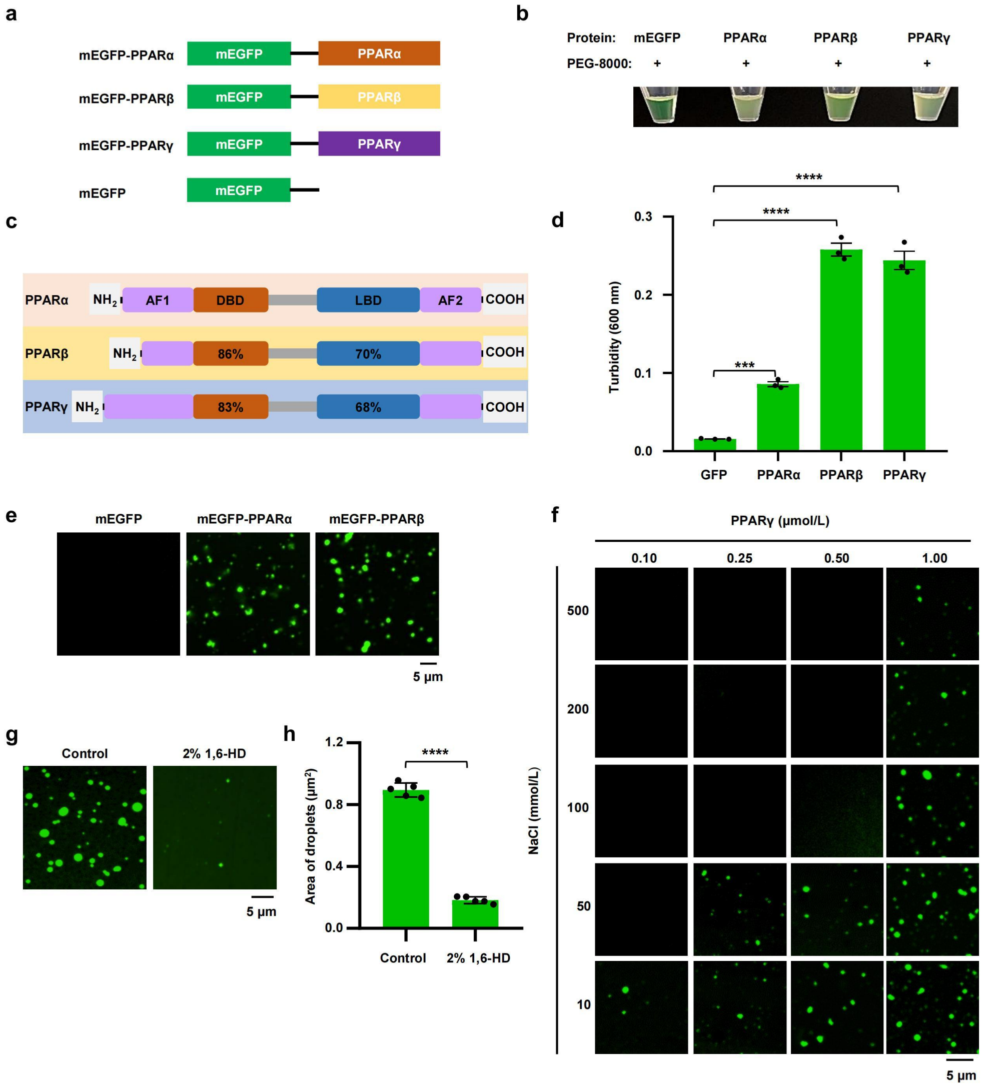

Supplementary Fig. S1 PPARs undergo phase separation in vitro. a Schematic of recombinant mEGFP fusion proteins used here. **b** Visualization of turbidity associated with droplet formation (10 μmol/L protein, 150 μmol/L NaCl, 10% PEG-8000). Tubes containing mEGFP, mEGFP-PPARα, mEGFP-PPARβ and mEGFP-PPARγ (from left to right). **c** PPARα, PPARβ and PPARγ contain same domains and share high sequence homology. AF1 domain, DBD, LBD and AF2 domain represent as indicated. The DBD and LBD domain exhibit high sequence identity between the three isoforms. The number inside DBD and LBD domain corresponds to amino acid sequence identity of PPARβ and PPARγ relative to PPARα. **d** Turbidity assay to quantify phase separation of PPARα, PPARβ and PPARγ (10 μmol/L protein, 150 μmol/L NaCl, 10% PEG-8000). OD600 was normalized to the measurement of mEGFP control. Data were shown as means ± SEM (n = 3). **e** Representative fluorescence microscopy images of mEGFP-PPARα and mEGFP-PPARβ (5 μmol/L protein, 150 μmol/L NaCl, 10% PEG-8000). Scale bar, 5 μm. **f**

Representative images of phase diagram related to Fig. 1c. **g** Representative fluorescence microscopy images of PPARγ with or without 2% 1,6-HD treatment (5 μmol/L protein, 150 μmol/L NaCl, 10% PEG-8000). Scale bar, 5 μm. **h** Column scatter charts display average droplet area of each image related to Supplementary Fig. S1g. Data are shown as means ± SEM (n = 5). One-way analysis of variance (ANOVA) for Supplementary Fig. S1d. Two-tailed unpaired t-test for Supplementary Fig. S1h. **P* < 0.05, ***P* < 0.01, ****P* < 0.001, *****P* < 0.0001. ns, not significant.

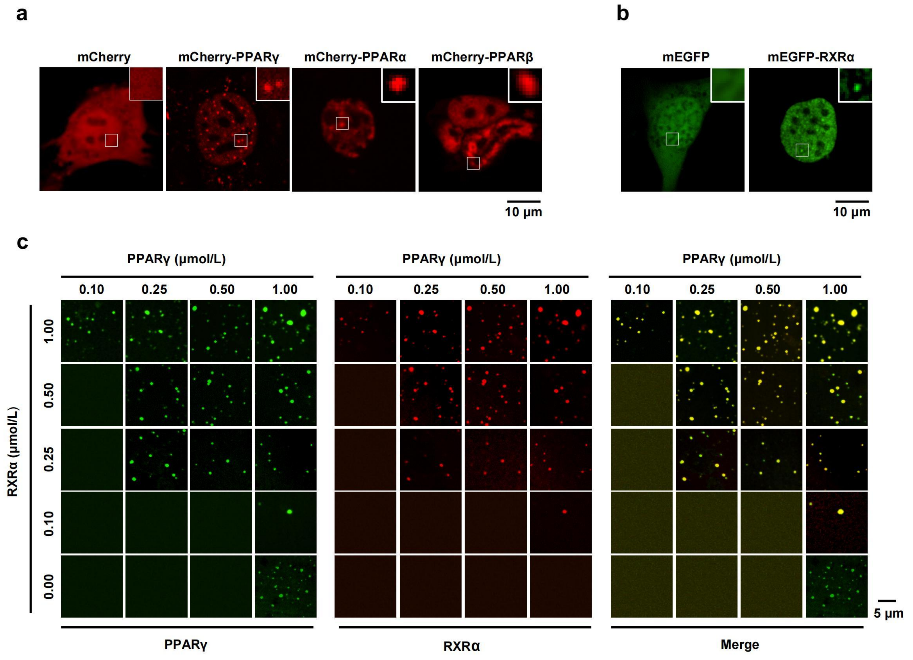

Supplementary Fig. S2 PPARs and RXRα undergo phase separation in vitro and in cells. a mCherry-PPARα, mCherry-PPARβ or mCherry-PPARγ formed puncta in 3T3-L1 cells. Cells were transfected with mCherry control, mCherry-PPARα, mCherry-PPARβ or mCherry-PPARγ for 48 h and imaged. **b** mEGFP-RXRα formed puncta in 3T3-L1 cells. Cells were transfected with mEGFP control or mEGFP-RXRα for 48 h and imaged. **c** Representative images of phase diagram related to Fig. 1j.

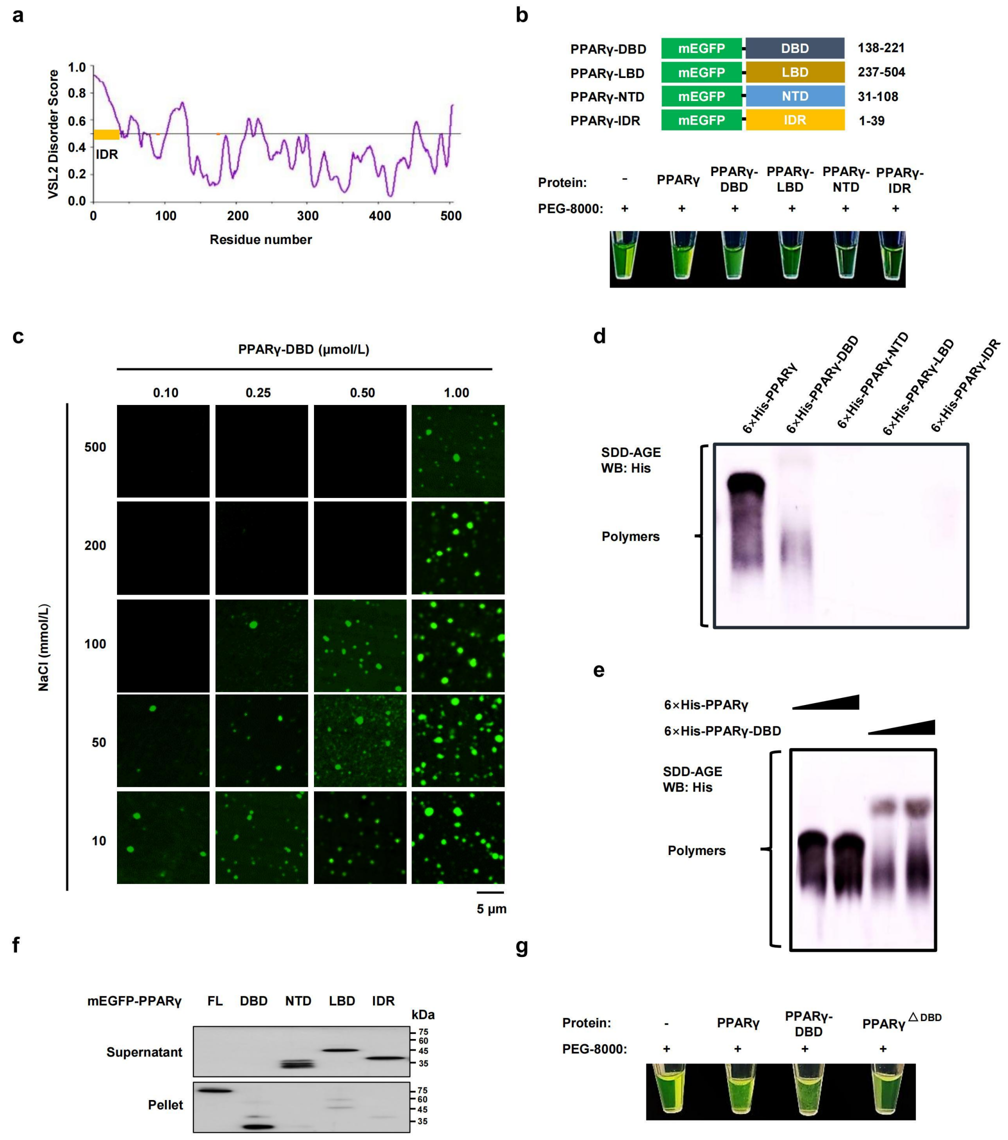

Supplementary Fig. S3 DBD is necessary for PPARγ phase separation. **a** Graphs plotting intrinsic disorder for different PPARγ function domains. PONDR VSL2 Score (y-axis) and amino acid position (x-axis) are as indicated. **b** Visualization of turbidity associated with droplet formation (10 μmol/L protein, 150 μmol/L NaCl, 10% PEG-8000). Tubes containing mEGFP, mEGFP-PPARγ, mEGFP-PPARγ-DBD, mEGFP-PPARγ-LBD, mEGFP-PPARγ-NTD and mEGFP-PPARγ-IDR (from left to right) are shown. **c** Representative images of phase diagram related to Fig. 2c. **d** SDD-AGE assay for different domains of indicated proteins. SDD-AGE assay is a method to detect protein aggregates. The purified proteins were diluted utilizing dilution buffer (50 mmol/L Tris-HCl, pH 7.5 and 500 mmol/L NaCl) to the volume of 10 μL and loaded with 6 × loading buffer. The amount of loaded protein is 2 μg and the used antibodies for Western blotting assays is anti-His. **e** SDD-AGE assay for different amounts of mEGFP-PPARγ and

mEGFP-PPARγ-DBD. The amount of loaded proteins is 2 μg and 500 ng, respectively. **f** Diagram of the sedimentation assay to separate the condensed phase and the aqueous phase of PPARγ and PPARγ truncations, followed by immunoblotting assays (10 μmol/L protein, 150 μmol/L NaCl, 10% PEG-8000). **g** Visualization of turbidity associated with droplet formation for mEGFP alone, mEGFP-PPARγ, mEGFP-PPARγ-DBD and mEGFP-PPARγ$^{\Delta DBD}$ (10 μmol/L protein, 150 μmol/L NaCl, 10% PEG-8000).

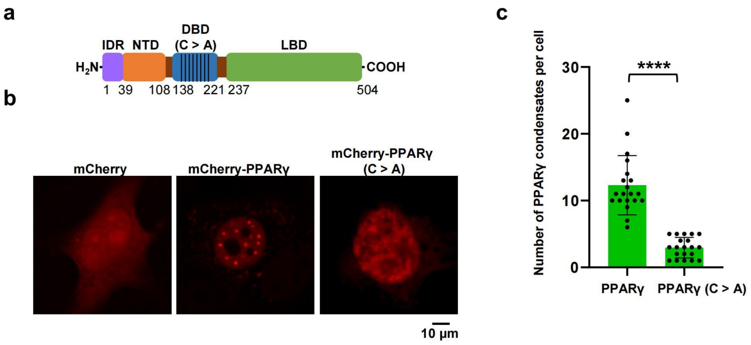

Supplementary Fig. S4 The disruption of zinc finger motif in DBD abolished the formation of PPARγ condensates in cells. **a** A schematic representation of PPARγ with the disruption of zinc finger motif in DBD. All the cysteines in zinc finger motif in DBD were mutated to alanines (PPARγ C > A). **b** Representative images of wild type PPARγ or PPARγ C > A mutant expressed in adipocytes. **c** The condensate number of wild type PPARγ or PPARγ C > A mutant in 3T3-L1 cells related to Supplementary Fig. S4b. Data are shown as means ± SEM (n = 20). Two-tailed unpaired t-test. *$P < 0.05$, **$P < 0.01$, ***$P < 0.001$, ****$P < 0.0001$. ns, not significant.

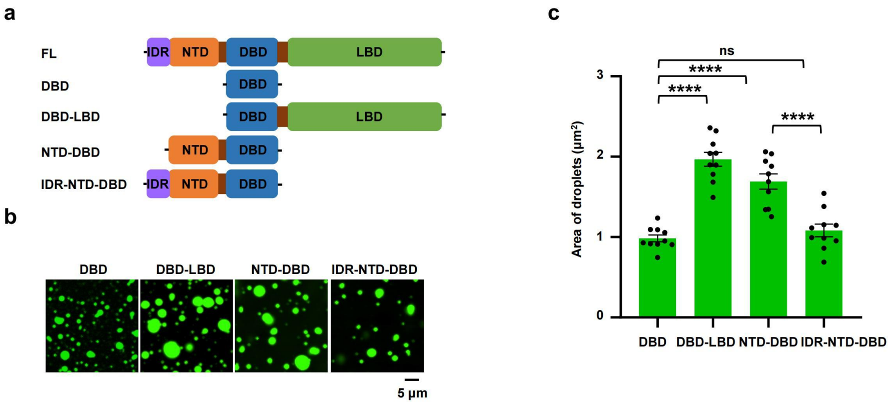

Supplementary Fig. S5 The intramolecular interaction between various domains regulated PPARγ phase separation. **a** The schematic diagram showing PPARγ truncations. **b**

Representative fluorescence microscopy images for phase separation of PPARγ-DBD fused to the other functional domains of PPARγ (5 μmol/L protein, 150 μmol/L NaCl, 10% PEG-8000). **c** Column scatter charts display average droplet area of each image related to Supplementary Fig. S5b. Data are shown as means ± SEM (n = 5). Two-tailed unpaired t-test. *P < 0.05, **P < 0.01, ***P < 0.001, ****P < 0.0001. ns, not significant.

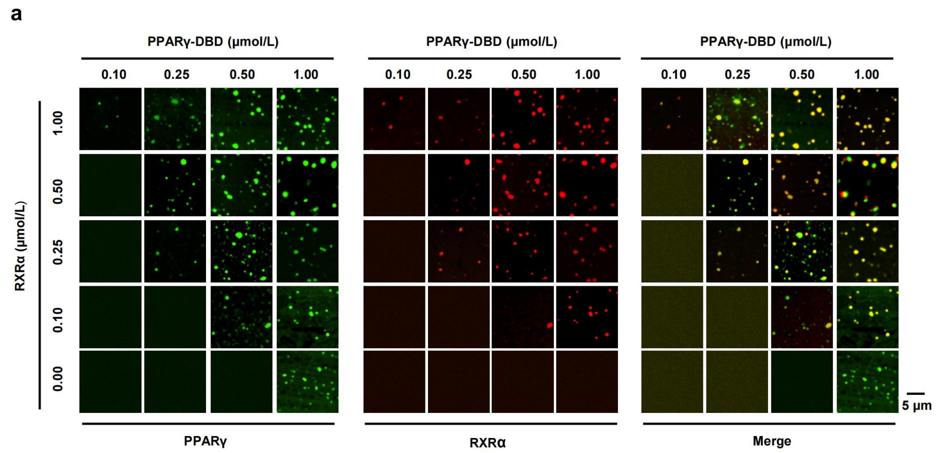

Supplementary Fig. S6 Representative images of phase diagram related to Fig. 2g.

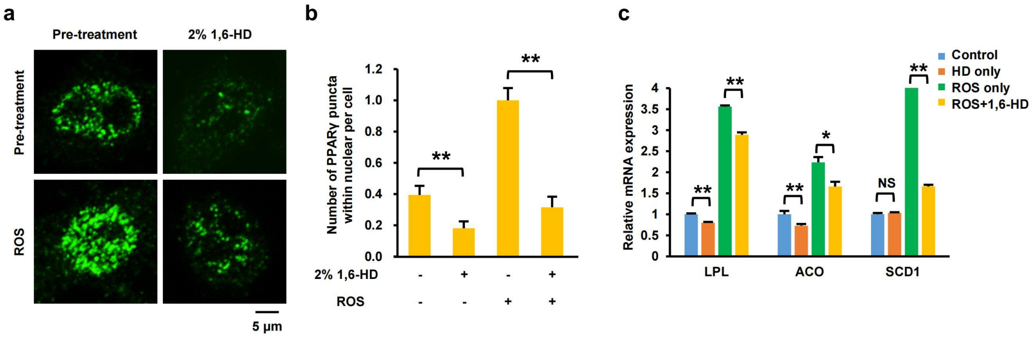

Supplementary Fig. S7 1,6-hexanediol (1,6-HD: 2%) disrupted droplet formation of PPARγ and impaired the mRNA expression of PPARγ target gene. a–b1,6-hexanediol (1,6-HD: 2%) disrupted droplet formation of PPARγ (n = 20). Data are shown as means ± SEM. Data were normalized to 100% by number of 3T3-L1 cells with only rosiglitazone treatment. Scale bar, 5 μm. ROS, rosiglitazone (50 μmol/L). **c** Relative mRNA expression of PPARγ target genes (LPL, ACO, SCD1) after 1,6-HD treatment. Data are shown as means ± SEM (n = 9). Two-tailed unpaired t-test. *P < 0.05, **P < 0.01, ***P < 0.001, ****P < 0.0001. ns, not significant.

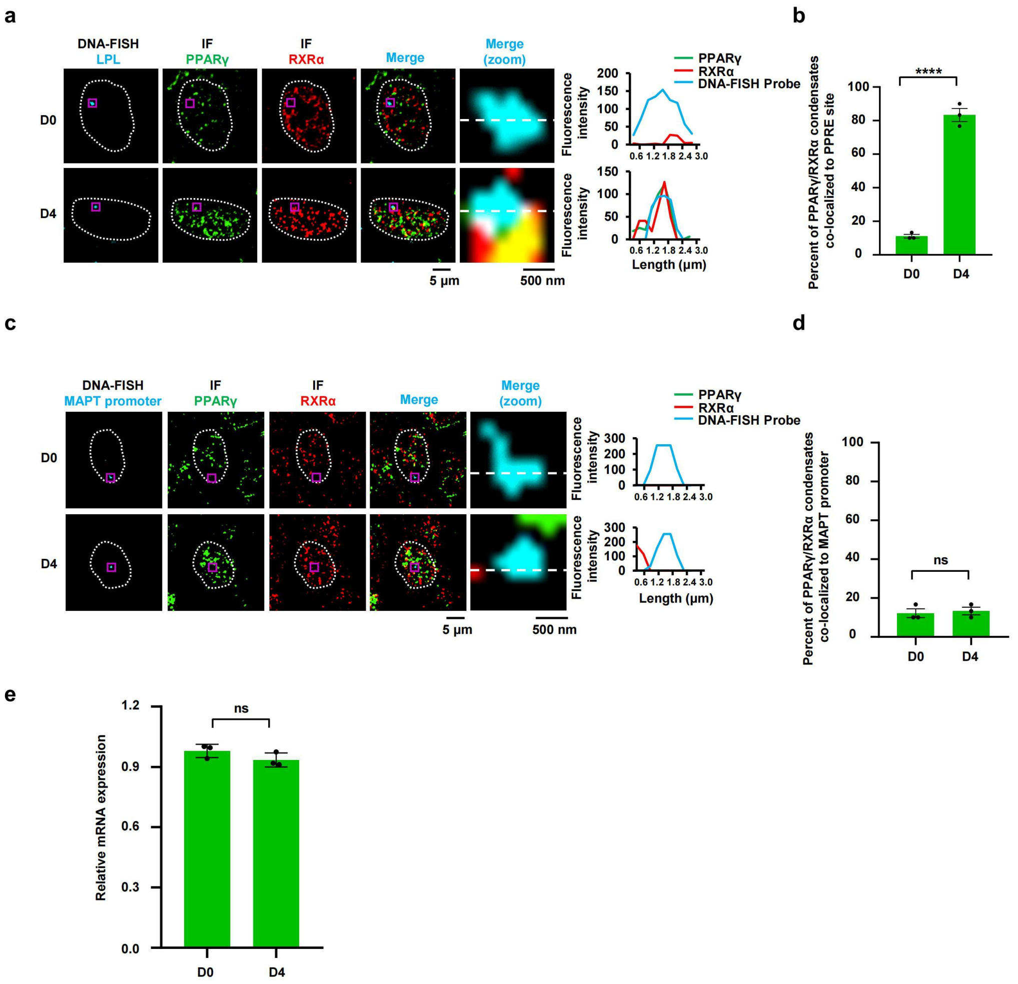

Supplementary Fig. S8 PPARγ/RXRα condensates specifically accumulated at PPREs before and after differentiation. a Co-localization between PPARγ/RXRα heterodimer condensates and LPL-PPRE loci by IF and DNA-FISH in fixed 3T3-L1 cells before and after differentiation. Separate images of the indicated LPL-PPRE probe (first column), PPARγ (second column) and RXRα (third column) are shown, accompanied with an image showing the merged channels (the fourth column, overlapping signal in white). Scale bar, 5 μm. The fifth column (merge (zoom)) displays a magnification of the purple box region in the fourth column for greater detail. Scale bar, 500 nm. The sixth column is then line plot corresponding to magnified image on the left. **b** Quantification of DNA-FISH analysis using percentage of cells with PPARγ/RXRα condensates and LPL-PPRE locus co-localization in fixed 3T3-L1 cells before and after differentiation ($n = 3$). Data are shown as means ± SEM. **c** Co-localization between PPARγ/RXRα heterodimer condensates and MAPT promoter loci by IF and DNA-FISH in fixed 3T3-L1 cells before and after differentiation. **d** Quantification of DNA-FISH analysis using percentage of cells with PPARγ/RXRα condensates and MAPT promoter loci co-localization in fixed 3T3-L1 cells before

and after differentiation (n = 3). Data are shown as means ± SEM. **e** RT-qPCR analysis of MAPT transcription levels in 3T3-L1 cells before and after differentiation (n = 3). Data are shown as means ± SEM. Two-tailed unpaired t-test. **P* < 0.05, ***P* < 0.01, ****P* < 0.001, *****P* < 0.0001. ns, not significant.

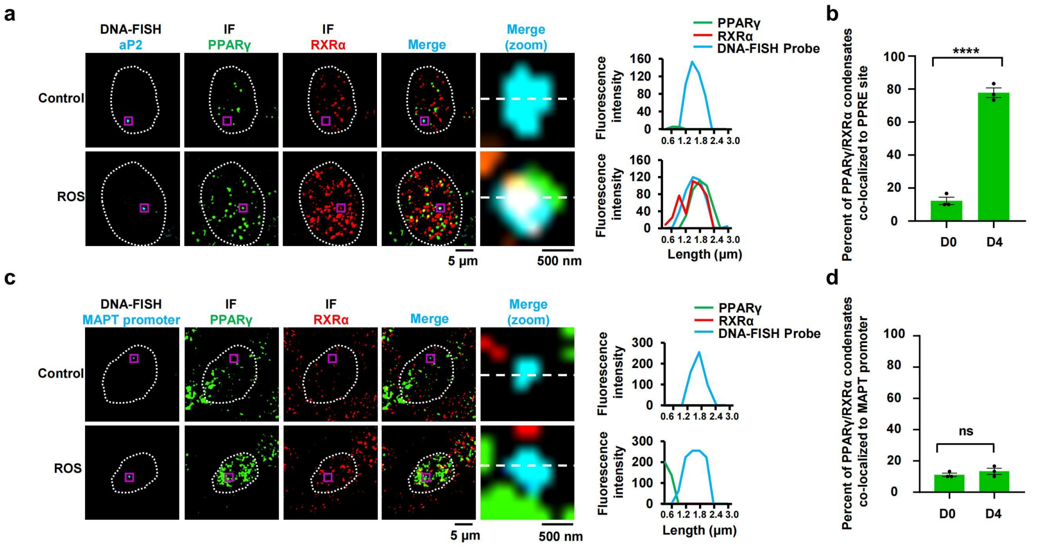

Supplementary Fig. S9 PPARγ/RXRα condensates specifically accumulated at PPREs before and after rosiglitazone treatment. **a** Co-localization between PPARγ/RXRα heterodimer puncta and aP2-PPRE locus by IF and DNA-FISH in fixed 3T3-L1 cells after rosiglitazone treatment. 3T3-L1 cells were seeded and treated with 50 μmol/L rosiglitazone for 48 h, and then fixed for IF and Fish immunofluorescence staining. Scale bar, 500 nm. **b** Quantification of DNA-FISH analysis using percentage of cells with PPARγ/RXRα condensates and aP2-PPRE locus co-localization in fixed 3T3-L1 cells before and after rosiglitazone treatment (n = 3). Data are shown as means ± SEM. **c** Co-localization between PPARγ/RXRα heterodimer condensates and MAPT promoter loci by IF and DNA-FISH in fixed 3T3-L1 cells before and after rosiglitazone treatment. **d** Quantification of DNA-FISH analysis using percentage of cells with PPARγ/RXRα condensates and MAPT promoter loci co-localization in fixed 3T3-L1 cells before and after rosiglitazone treatment (n = 3). Data are shown as means ± SEM. Two-tailed unpaired t-test. **P* < 0.05, ***P* < 0.01, ****P* < 0.001, *****P* < 0.0001. ns, not significant.

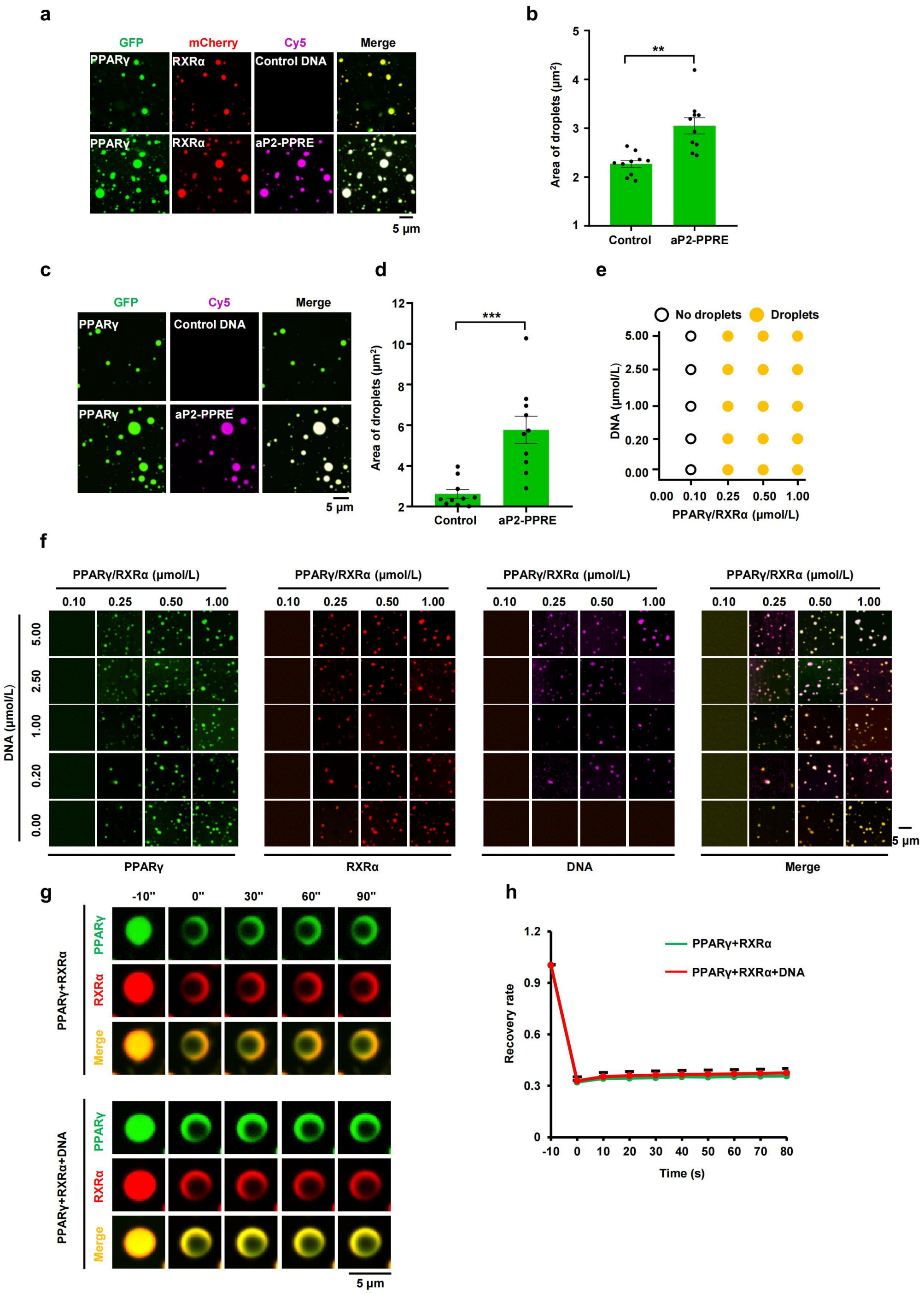

Supplementary Fig. S10 The PPARγ/RXRα droplets specifically phase separated with PPRE. **a** Representative fluorescence microscopy images of a mixture of mEGFP-PPARγ/mCherry-RXRα with 5 μmol/L DNAs encoding control sequence or aP2-PPRE sequence (1 μmol/L protein, 150 μmol/L NaCl, 10% PEG-8000). Scale bar, 5 μm. **b** Column scatter charts display average droplet area of each image in reactions related to Supplementary Fig. S10a. Data are shown as means ± SEM (n = 10). **c** Representative fluorescence microscopy

images of a mixture of mEGFP-PPARγ with 5 μmol/L DNAs encoding random sequence or aP2-PPRE sequence (1 μmol/L protein, 150 μmol/L NaCl, 10% PEG-8000). Scale bar, 5 μm. **d** Column scatter charts display average droplet area of each image related to Supplementary Fig. S10c. Data are shown as means ± SEM (n = 10). **e** Phase diagram of PPARγ/RXRα complex in the presence of different concentration of PPRE. **f** Representative images of phase diagram related to Supplementary Fig. S10e. **g** FRAP analysis of PPARγ/RXRα mixture with or without 5 μmol/L DNA sequence of aP2-PPRE. Scale bar, 5 μm. **h** Quantification of changes in the fluorescence measurement were plotted over time. The background was subtracted from the fluorescence measurement. Values represent means ± SEM (n = 15). Two-tailed unpaired t-test for Supplementary Fig. S10b and Supplementary Fig. S10d. **P* < 0.05, ***P* < 0.01, ****P* < 0.001, *****P* < 0.0001. ns, not significant.

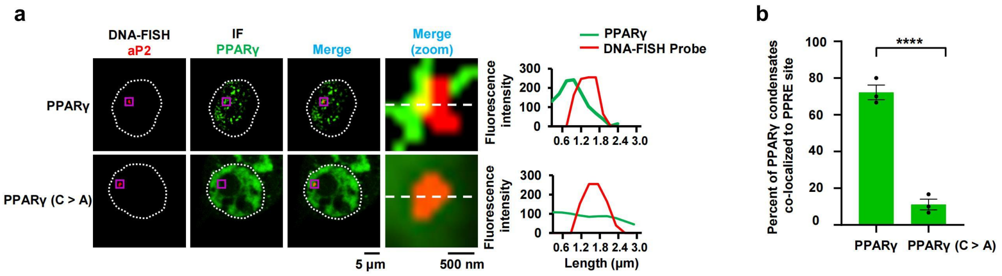

Supplementary Fig. S11 The PPARγ with the disruption of zinc finger motif failed to enrich at PPRE site. a Co-localization between PPARγ WT or PPARγ (C > A) mutant and aP2-PPRE locus by IF and DNA-FISH in fixed 3T3-L1 cells after ROS treatment. **b** Quantification of DNA-FISH analysis using percentage of cells with PPARγ WT or PPARγ (C > A) mutant and aP2-PPRE locus co-localization in fixed 3T3-L1 cells before and after rosiglitazone treatment (n = 3). Data are shown as means ± SEM. Two-tailed unpaired t-test. **P* < 0.05, ***P* < 0.01, ****P* < 0.001, *****P* < 0.0001. ns, not significant.

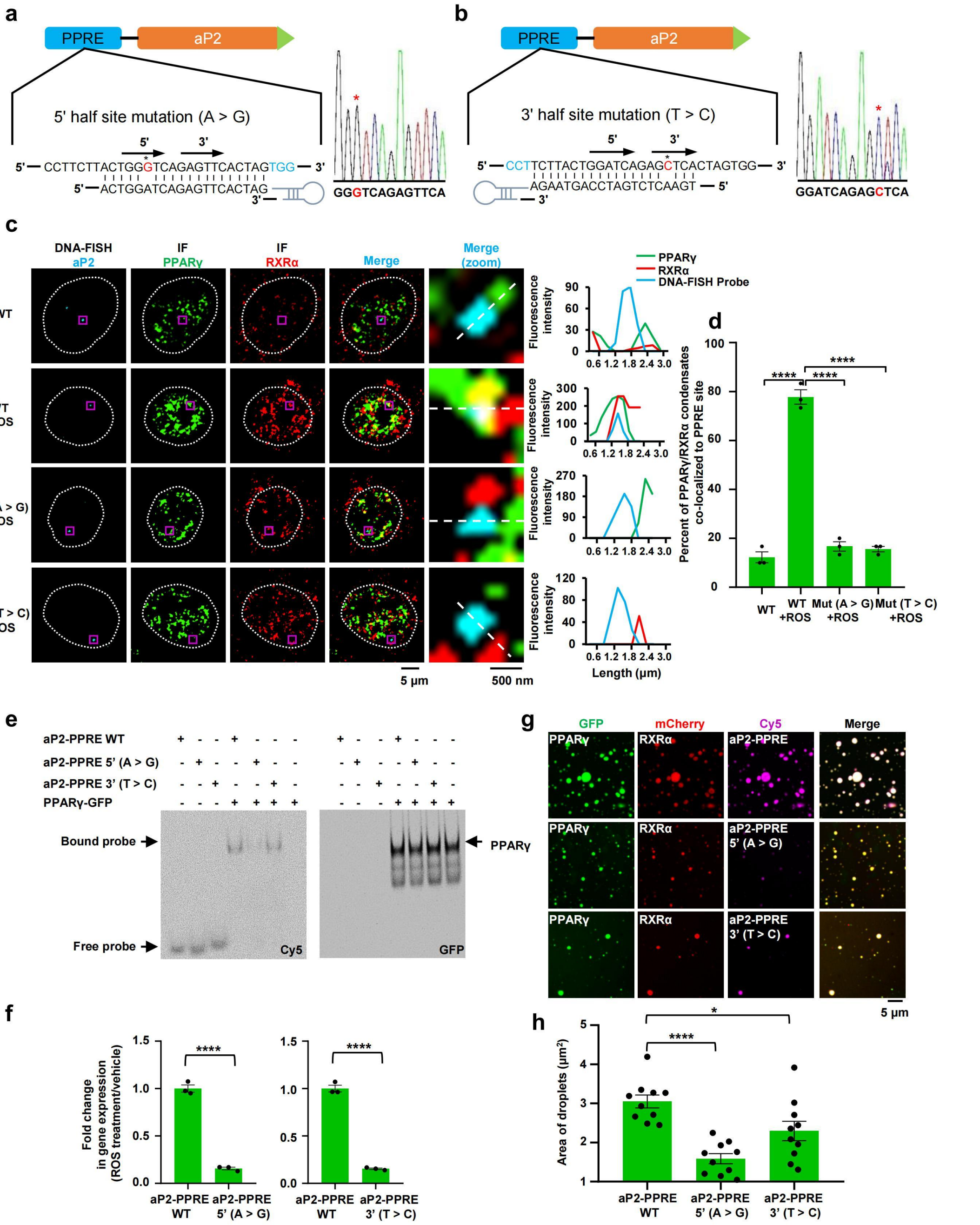

Supplementary Fig. S12 PPRE mutation impaired mRNA expression of PPARγ target gene when PPARγ/RXRα condensates failed to localize at PPRE sites. a – b Base editing-induced aP2-PPRE mutation in 3T3-L1 cells. Left, Schematic view of aP2-PPRE domains and base editor target site. The base mutation in aP2-PPRE domains is labeled in red. PAM sequences is indicated in blue. Green arrow indicates the direction of transcription. Right, DNA sequence analysis of aP2-PPRE domains after base editing. **c** Co-localization between PPARγ/RXRα heterodimer puncta and aP2-PPRE locus by IF and DNA-FISH in fixed 3T3-L1 cells (wild-type (WT) or PPRE mutation (Mut)) before and after rosiglitazone treatment. Separate images of the indicated

aP2-PPRE probe (first column), PPARγ (second column) and RXRα (third column) are shown, accompanied with an image showing the merged channels (the fourth column, overlapping signal in white). Scale bar, 5 μm. The fifth column (merge (zoom)) displays the magnification of the purple box region in the fourth column for greater detail. Scale bar, 500 nm. The sixth column is the line plot corresponding to magnified image. ROS, 50 μmol/L. **d** Quantification of DNA-FISH analysis using percentage of cells with PPARγ/RXRα condensates and aP2-PPRE loci (WT or Mut) co-localization in fixed 3T3-L1 cells upon rosiglitazone treatment ($n = 3$) **e** The interaction between PPARγ and PPRE measured by electrophoretic mobility shift assay (EMSA). **f** mRNA fold change measured by qRT-PCR for aP2 gene of WT and Mut (A > G and T > C) after rosiglitazone treatment on 3T3-L1 cells. ROS, 50 μmol/L. Data are normalized to WT cells. Data are shown as means ± SEM ($n = 9$). **g** Representative fluorescence microscopy images of a mixture of mEGFP-PPARγ/mCherry-RXRα with 5 μmol/L DNA of aP2-PPRE, aP2-PPRE 5' (A > G) or aP2-PPRE 3' (T > C) respectively. The concentration of each protein in buffer is 1 μmol/L. Scale bar, 5 μm. **h** Column scatter charts display the droplet diameter in reactions related to Supplementary Fig. S12g. Data are shown as means ± SEM ($n = 10$). Two-tailed unpaired t-test for Supplementary Fig. S12f. One-way analysis of variance (ANOVA) for Supplementary Fig. S12d and Supplementary Fig. S12h. *$P < 0.05$, **$P < 0.01$, ***$P < 0.001$, ****$P < 0.0001$. ns, not significant.

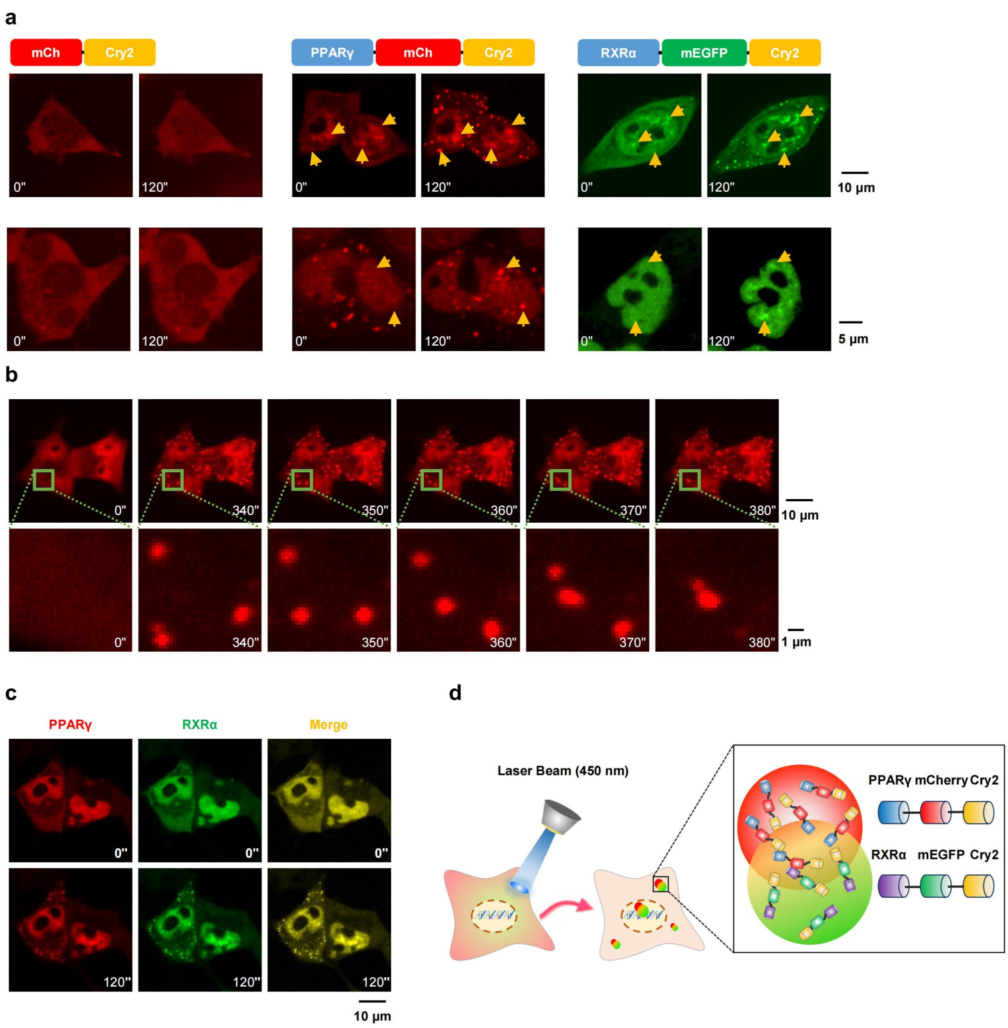

Supplementary Fig. S13 Light-induced optoPPARγ/optoRXRα heterodimer phase separation. **a** Images of cells expressing mCherry-Cry2, PPARγ-mCherry-Cry2 or RXRα-mEGFP-Cry2 (top: HEK293T cell, bottom: pre-3T3-L1 cell). Cells were subjected to blue light stimulation for 2 min. **b** Time-lapse images of HEK293T cells stably expressing PPARγ-mCherry-Cry2 exposed to blue light every ten seconds for the time indicated (top). Scale bar, 10 μm. The bottom images display the magnification of the green box region in the top image for higher resolution. Scale bar, 1 μm. **c** Images of HEK293T cells expressing PPARγ-mCherry-Cry2/RXRα-mEGFP-Cry2 exposed to blue light stimulation for 2 min. Separate images of the indicated PPARγ-mCherry-Cry2 (first column), RXRα-mEGFP-Cry2 (second column) are shown, accompanied with an image showing the merged channels (the third column, overlapping signal in yellow). Scale bar, 10 μm. **d** Schematic diagram for optoPPARγ/optoRXRα phase separation.

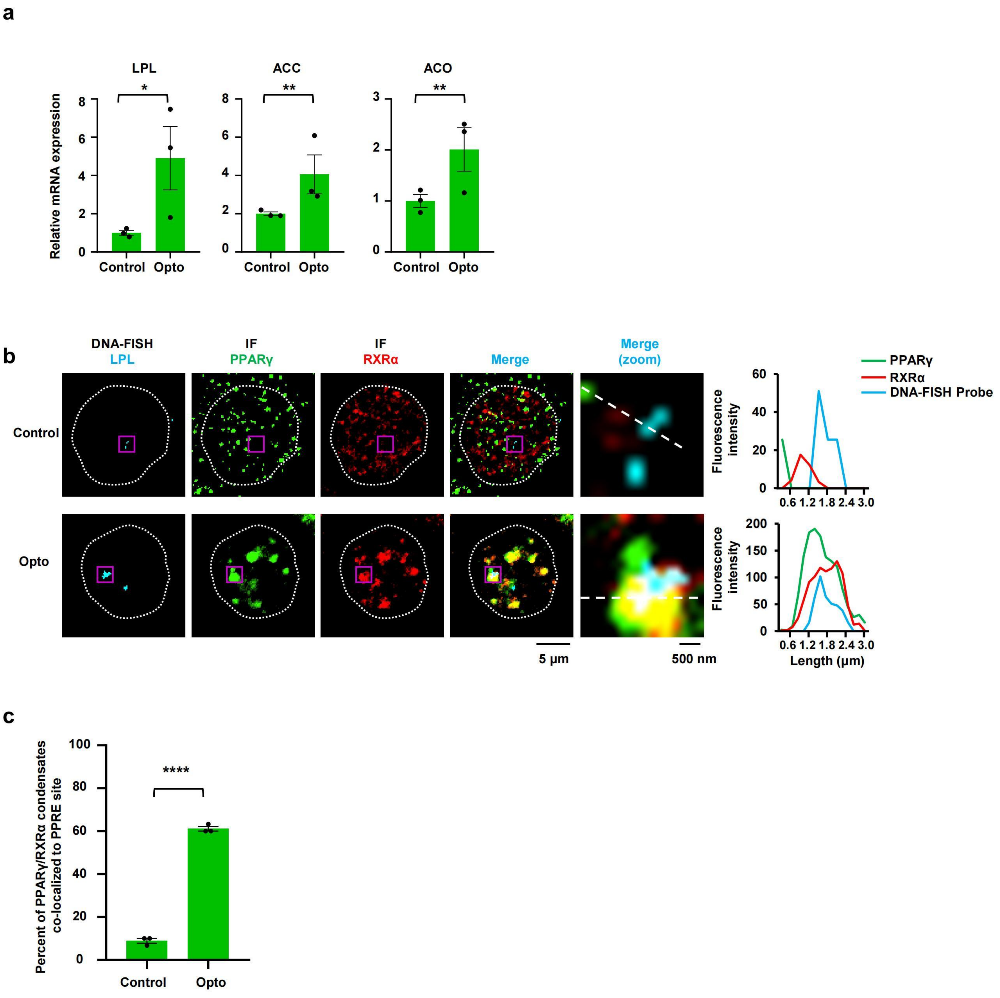

Supplementary Fig. S14 Light-induced optoPPARγ/optoRXRα heterodimer condensates prefer to be enriched at PPRE sites to promote target gene expression in HEK293T cells. a RT-qPCR analysis of LPL, ACC and ACO transcript levels in dark treated cells and light-treated cells. HEK293T cells with stable expression of PPARγ-mCherry-Cry2 and RXRα-mEGFP-Cry2 were seeded in the dish for 12 h and were treated by blue light for 24 h. Dark treated cells were referred as Control, and blue light treated cells were referred as Opto. Data are shown as means ± SEM (n = 9). **b** Co-localization between optoPPARγ/optoRXRα heterodimer puncta and LPL-PPRE locus by IF and DNA-FISH in fixed Control-HEK293T and Opto-HEK293T cell. Separate images of the indicated LPL-PPRE probe (first column), PPARγ (second column) and RXRα (third column) are shown, accompanied with an image showing the merged channels (the fourth column, overlapping signal in white). Scale bar, 5 μm. The fifth column (merge (zoom)) displays then magnification of the purple box region in the fourth column for greater detail. Scale bar, 500 nm. The sixth column is the line plot corresponding to magnified image. **c** Quantification

of DNA-FISH analysis using percentage of cells with PPARγ/RXRα condensates and LPL-PPRE locus co-localization in fixed in Control-293T and Opto-293T cell ($n = 3$). Data are shown as means ± SEM. Two-tailed unpaired t-test. *$P < 0.05$, **$P < 0.01$, ***$P < 0.001$, ****$P < 0.0001$. ns, not significant.
